# Phase Separation of Aqueous Poly(diisopropylaminoethyl methacrylate) upon Heating

**DOI:** 10.1021/acs.langmuir.1c02224

**Published:** 2021-11-09

**Authors:** Linda Salminen, Erno Karjalainen, Vladimir Aseyev, Heikki Tenhu

**Affiliations:** † Department of Chemistry, University of Helsinki, P.O. Box 55, A.I. Virtasen aukio 1, FIN-00014 HY Helsinki, Finland; ‡ VTT Technical Research Centre of Finland Ltd., P.O. Box 1000, FI-02044 VTT Espoo, Finland

## Abstract

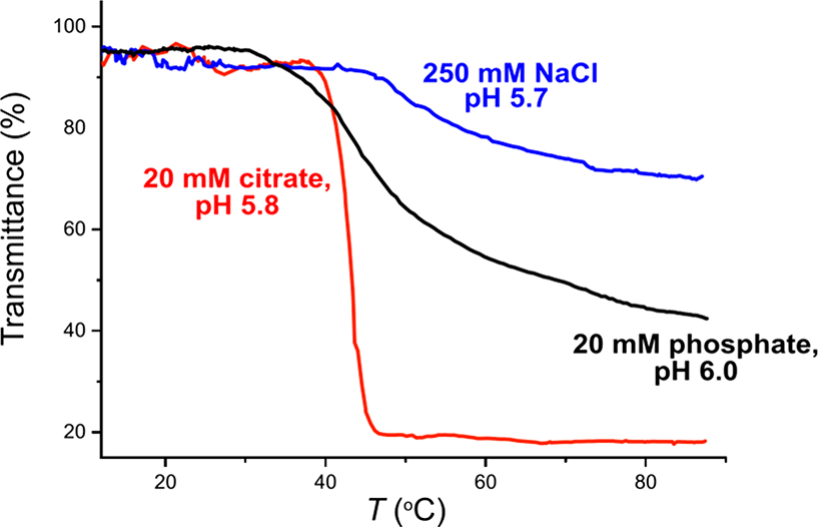

Poly(diisopropylaminoethyl methacrylate) (PDPA) is a pH- and thermally responsive water-soluble polymer. This study deepens the understanding of its phase separation behavior upon heating. Phase separation upon heating was investigated in salt solutions of varying pH and ionic strength. The effect of the counterion on the phase transition upon heating is clearly demonstrated for chloride-, phosphate-, and citrate-anions. Phase separation did not occur in pure water. The buffer solutions exhibited similar cloud points, but phase separation occurred in different pH ranges and with different mechanisms. The solution behavior of a block copolymer comprising poly(dimethylaminoethyl methacrylate) (PDMAEMA) and PDPA was investigated. Since the PDMAEMA and PDPA blocks phase separate within different pH- and temperature ranges, the block copolymer forms micelle-like structures at high temperature or pH.

## Introduction

Stimuli-responsive polymers have been the focus of many studies due to their potential applications in, e.g., drug delivery^1−5^ and actuators.^6−10^ Of all potential triggers, temperature and pH are of special interest. Thermally responsive polymers are generally divided into those with the lower critical solution temperature (LCST; phase separation upon heating) behavior and those with the upper critical solution temperature (UCST; phase separation upon cooling) behavior, although variations exist within the two classes.^11^

Phase separation upon heating aqueous polymers derives from entropy changes of water molecules. At low temperatures, the polymer is in a coiled form and the surrounding water molecules are in an energetically favored but ordered state. As the temperature increases, the entropic contribution overrules the energetic advantages of the ordered state and the interactions between water molecules and the polymer weaken. Consequently, the hydrophobic backbone of the polymer starts to interact more strongly with other nonpolar moieties, forming a macroscopic precipitate.^12−17^

Polymers that phase separate upon cooling in aqueous solutions are less common than the ones that phase separate upon heating.^18,19^ Phase transitions that occur upon cooling are defined by strong supramolecular attraction of the polymer chains, i.e., electrostatic bonds or hydrogen bonds.^11^ These supramolecular interactions weaken upon heating, solubilizing the polymer.^20^ In contrast to phase separation upon heating that is mainly driven by entropy, the phase separation upon cooling is driven by enthalpy.^21,22^

Poly(dialkylaminoethyl methacrylate)s are polymers that consist of tertiary aminemethacrylate monomers. The best-known one is poly(dimethylaminoethyl methacrylate) (PDMAEMA). PDMAEMA is a weak cationic polyelectrolyte, which is soluble in water in neutral and acidic solutions.^23^ Double stimuli-responsive polymers have potential uses in many applications, since the presence of multiple sensitivities makes subtle and well-controlled conformation adjustments possible.^24^ PDMAEMA, for instance, responds to changes in both temperature and pH^25,26^ and is thus very promising for applications in, e.g., drug delivery,^27−29^ antimicrobial or antifogging membranes,^30−33^ and actuators.^34,35^ Double stimuli-responsive homopolymers are few in number, and therefore additional sensitivities have been introduced through copolymerization. However, the fastidious, multistep processes in synthesizing block copolymers make homopolymers or random copolymers more advantageous.

Poly(diisopropylaminoethyl methacrylate) (PDPA) only differs from PDMAEMA in the alkyl substituents of the amine group. Despite its close resemblance to PDMAEMA, the phase transition behavior of PDPA is relatively less known. PDPA has been regarded mainly as a pH-sensitive polymer, and only a few studies have reported the polymer as both pH- and thermoresponsive.^26,36^ Thavanesan et al. have shown that PDPA phase separates upon heating in a narrow pH range.^36^ Their study included PDMAEMA, PDPA, as well as poly(diethylaminoethyl methacrylate) (PDEAEMA). They concluded that all three polymers were responsive to both pH and temperature. They pointed out that the pH ranges of the transitions did not align with the polymers’ p*K*_a_ values (p*K*_a_(PDMAEMA) ≈ 6.2, p*K*_a_(PDEAEMA) ≈ 6.7, p*K*_a_(PDPA) ≈ 6.9).^36^ Instead, the pH- and temperature-ranges where LCST behavior was observed were mainly affected by the size of the dialkylaminoethyl group. That is, p*K*_a_ increases with increasing hydrophobicity of the substituent. This is unexpected since polyamines have been reported to exhibit lower p*K*_a_ values with more hydrophobic substituents.^37^ It should be noted that others report the p*K*_a_ values of the same polymers to decrease with increasing hydrophobicity of the amine substituents, giving respective p*K*_a_ values of 7.0, 7.3, and 6.0 for PDMAEMA, PDEAEMA, and PDPA.^23,38^

The pH and temperature required for the LCST type transition decrease with increasing size and hydrophobicity of the dialkylaminoethyl substituent. In addition, Thavanesan et al. found out that the phase separation of PDMAEMA was highly dependent on the interactions between the carbonyl group and polymer backbone, whereas the phase separations of PDEAEMA and PDPA were dictated by their diethylaminoethyl and diisopropylaminoethyl groups. In the case of PDPA, the diisopropylaminoethyl substituent tends to twist toward the polymer backbone in order to minimize its contact to water even in the soluble state of the polymer.^36^

This contribution delves into the phase separation behavior of PDPA. The effects of various salts, the ionic strength, and pH on the phase separation temperature are reported. The studied salt anions, citrate, monohydrogen phosphate, and chloride, have valences of 3, 2, and 1. Counterion valency is known to affect the collapsing behavior of branched polyelectrolytes and polyelectrolyte brushes. Compared to monovalent salt ions, multivalent counterions require lower concentrations to collapse polyelectrolyte brushes.^39−41^ The phase separation behavior is investigated using turbidimetry, microcalorimetry, light scattering, and fluorescence. In addition to the PDPA homopolymer, PDMAEMA-*b*-PDPA block copolymer is studied, and the obtained results are presented. PDMAEMA and PDPA have similar stimuli-sensitivities, which appear in different pH-ranges.^36^ Due to their distinct solubility-ranges, block copolymers comprising PDMAEMA and PDPA form micelles as a response to changes in pH.^23,42^ Past research has only noted the effects of pH, whereas this study also addresses heat-induced micellization.

Deeper understanding of the solution behavior of PDPA is beneficial, since double stimuli-responsive homopolymers are uncommon. Because PDPA responds to both pH and temperature, it has potential to be utilized in a wider variety of applications. The current usage of PDPA is limited to the utilization of the polymer’s response to changing the pH.^38,43−45^

## Experimental Section

### Materials

2-(Diisopropylamino)ethyl methacrylate (DPA) (Aldrich, 97%) and 2-(dimethylamino)ethyl methacrylate (DMAEMA) (Acros Organics, 99%) were passed through basic Al_2_O_3_ and distilled under reduced pressure. Azobis(isobutyronitrile) (AIBN) (Fluka, 98%) was recrystallized from methanol. Toluene (Merck, HPLC grade) was distilled. The chain transfer agent 4-cyano-4-(phenylcarbonothioylthio)pentanoic acid (CPA) (Aldrich, 97%), HCl solution (FF-Chemicals), NaOH solution (FF-Chemicals), citrate buffer with pH 4 (Fluka), phosphate buffer with pH 7 (VWR), carbonate buffer with pH 10 (VWR), trisodium citrate (BDH Chemicals), pyrene (Fluka), disodium hydrogen phosphate 2-hydrate (Applichem, ≥ 99%), sodium sulfate (Merck, ≥99%), tetrabutylammonium bromide (Aldrich, ≥98%), sodium tetraborate decahydrate (Fluka, ≥99.5%), and NaCl (Fisher Scientific, analytical reagent grade) were used as received.

### Syntheses

#### Poly(diisopropylaminoethyl methacrylate) (PDPA)

The polymer was synthesized by the reversible addition–fragmentation chain transfer (RAFT) polymerization method. In a flask, 0.0330 g (0.118 mmol) of CPA and 0.0020 g (0.0122 mmol) of AIBN were dissolved in 5.0074 g (23.47 mmol) of diisopropylaminoethyl methacrylate. The mixture was purged with nitrogen for 30 min. Then the mixture was allowed to react at 90 °C under a nitrogen atmosphere. After 17 h, the polymerization was stopped by immersing the flask in liquid nitrogen. A sample was taken for determining the conversion of the reaction. The mixture was dissolved in acetone with a small amount of dichloromethane, and the polymer was precipitated to acetonitrile. This was repeated three times. The polymer was then dried under vacuum.

#### Poly(dimethylaminoethyl methacrylate) (PDMAEMA)

PDMAEMA was also synthesized using the RAFT method. In a flask, 0.0297 g (0.106 mmol) of CPA and 0.00175 g (0.01065 mmol) of AIBN were dissolved into 5.00001 g (31.80 mmol) of dimethylaminoethyl methacrylate. The mixture was purged with nitrogen for 30 min, and then the mixture was let to react at 90 °C for 17 h. The polymerization was stopped by immersing the flask in liquid nitrogen. At this point, a sample was taken for determining the conversion of the reaction. The polymer was purified by precipitating from acetone to cold hexane. The polymer was further purified by dialysis against water for 6 days, changing the water twice a day. The polymer was isolated by freeze-drying.

#### PDMAEMA-*b*-PDPA

A block copolymer was synthesized using PDMAEMA as a macrochain transfer agent. In total, 1.00049 g (6.39 mmol of repeating units) of PDMAEMA, 0.69320 g (3.25 mmol) of DPA, and 0.00048 g (0.00292 mmol) of AIBN were dissolved in 5 mL of distilled toluene. A sample was taken for determining the conversion. The mixture was purged with nitrogen for a half hour. Then the flask was moved to an oil bath (90 °C), and it was left to react for 17 h. The reaction was stopped by immersing the flask in liquid nitrogen, and a sample was taken for determining the conversion. Toluene was evaporated off. The polymer was precipitated from acetone to cold hexane. The precipitate was dissolved in methanol, and the polymer was further purified by dialysis against water for 9 days, changing the water twice a day. The polymer was isolated by freeze-drying.

### Methods

#### Size Exclusion Chromatography

The molar masses of all synthesized polymers were determined with size-exclusion chromatography (SEC). The equipment consisted of a Waters 515 HPLC pump, three Waters Styragel capillary columns, a Viscotek 270 dual light scattering and viscosity detector, a Waters 2487 UV detector, and a Waters 2410 refractive index (RI) detector. The system was calibrated using poly(methyl methacrylate) standards, and THF with 0.1% tetrabutylammonium bromide and 1% toluene was used as the eluent.

#### Proton Nuclear Magnetic Resonance (^1^H NMR)

^1^H NMR spectra were recorded with a Bruker Avance III 500 spectrometer to determine conversions and to ascertain the purity of the products.

#### Transmittance Measurements

Transmittance as a function of temperature was measured with a JASCO J/815 CD spectrometer. The transmittances of the samples were monitored at the wavelength of 600 nm. The measurements were conducted in 10 mm cuvettes, and the samples were degassed in vacuum before the measurements. The transmittances of the samples were measured from 5 to 90 °C with 1 °C/min heating and cooling rates. The samples were let to equilibrate in the starting temperature for 10 min before starting the measurement. The cooling run was started immediately after the heating was completed. The transmittance of the pure solvent was set to be 100%, and the volume of the sample was always 2.80 mL. The cloud point was defined to be the onset determined by the intersection of two tangents from the transmittance curve obtained during the heating cycle (see Figure S1a). The clearing point, the temperature where the solution cleared upon cooling, was defined analogously from the cooling run.

#### pH Measurements

The pH of the solutions was measured with a VWR pHenomenal IS 2100L pH meter. The electrode was calibrated using a citrate buffer with pH 4, phosphate buffer with pH 7, and a carbonate buffer with pH 10.

#### Microdifferential Scanning Calorimetry

Microdifferential scanning calorimetry (microDSC) measurements were conducted with a Malvern MicroCal PEAQ-DSC microcalorimeter. The heat of the sample was measured relative to pure water, and the enthalpy values were normalized to the molar concentration of the repeating unit. Like in transmittance measurements, the samples were degassed at 5 °C prior to measurements. Each sample was heated with a rate of 1 °C/min from 5 to 100 °C. The samples were equilibrated for 30 min at the starting temperature prior to measurement. The cooling back to 5 °C was done with the same rate. The temperature of maximum heat capacity (*T*_max_), the starting point of transition (*T*_onset_), and the enthalpy change of the phase transition (Δ*H*) were determined from the thermograms. *T*_onset_ was defined as the intersection of two tangents. The locations of the tangents were chosen based on the derivative of the thermogram. The first tangent was drawn at the point where the heat flow first started to increase and the second where the slope was the steepest (Figure S1b).

#### Dynamic Light Scattering (DLS)

A Malvern Zetasizer Nano was used to measure light scattering at an angle of 173°. The sample was equilibrated at the initial temperature for 15 min prior to heating. Light scattering was measured every 2 degrees upon heating up to 90 °C. The sample was equilibrated before every measurement for 5 min.

#### UV–Visible Absorbance Measurements

Absorbance spectra were recorded using a Shimadzu UV-1601PC spectrophotometer. The measurements were carried out at 20 °C.

#### Fluorescence Measurements

Fluorescence measurements were conducted with a Horiba Jobin Yvon Fluoromax-4 spectrofluorometer. The measurements were performed at the range of 15–80 °C. The sample was equilibrated for 30 min at the starting temperature prior to measurement and 10 min in each measurement point. The excitation wavelength was 325 nm, and emission was monitored in the range of 350–600 nm.

### Preparation of Polymer Solutions

All samples were diluted from 10 mg/mL polymer solution. The stock solution was prepared by dissolving 250 mg of PDPA in a small amount of 150 mM hydrochloric acid (HCl) and diluting to 25 mL with 150 mM HCl. The stock solutions were prepared at least 1 day before measurements and stored refrigerated at 4 °C.

#### Transmittance- and DSC-Measurements

The polymer concentration was 2 mg/mL, and the solutions were prepared with citrate, phosphate, sulfate, or NaCl. The sample preparation was started by adding aqueous salt solution to the sample vial. The amount of salt solution was set so that the final salt concentration in a 3.00 mL sample would be 20 mM for the citrate-, sulfate-, and phosphate-solutions and 250 mM or 500 mM for the NaCl-solutions. Then, aqueous NaOH or HCl and water were added so that the sum of the additions was 2.40 mL. The amount of added NaOH or HCl depended on the aimed pH. Finally, 0.60 mL of the PDPA stock solution was added under vigorous stirring. The pH of the samples was measured immediately before measurements at room temperature.

Solutions were prepared at various pHs in the range of 4.5–6.5. It should be noted that since the adjustment of pH was done by varying the amounts of NaOH and HCl in the solution, even the solutions without added NaCl contain chloride anions and the concentration of Cl^–^ varies with the pH. However, the concentration of chloride is small compared to the other salt, that is, citrate, phosphate, or sulfate.

#### Fluorescence Measurements

The polymer concentration was 2 mg/mL, and solutions with citrate, phosphate, and NaCl were studied. Similar to the solutions used in transmittance measurements, the sample preparation was started by adding the salt solution to a vial. The salt concentrations were 20 mM for citrate, 20 mM for phosphate, and 250 mM for NaCl. A volume of 1.00 mL of saturated pyrene solution was added into the salt solution. Then, pure water and aqueous NaOH were added so that the sum of the additions was 2.40 mL. Finally, 0.60 mL of PDPA stock solution was added under vigorous stirring.

#### Dynamic Light Scattering

The sample preparation at pH 6 was the same as for the samples for transmittance- and DSC-measurements described above. At pH 8, PDMAEMA and PDMAEMA-*b*-PDPA were studied in a phosphate solution and at pH 10 in a borate solution. The phosphate solutions were prepared from sodium phosphate and the borate solutions from sodium tetraborate. The same sample preparation method was used as with the transmittance measurements: the aqueous salt, NaOH, and water were added so that the sum of the additions was 2.40 mL. Then 0.60 mL of polymer stock solution was added under vigorous stirring.

## Results and Discussion

### Polymerizations

**Chart 1:**
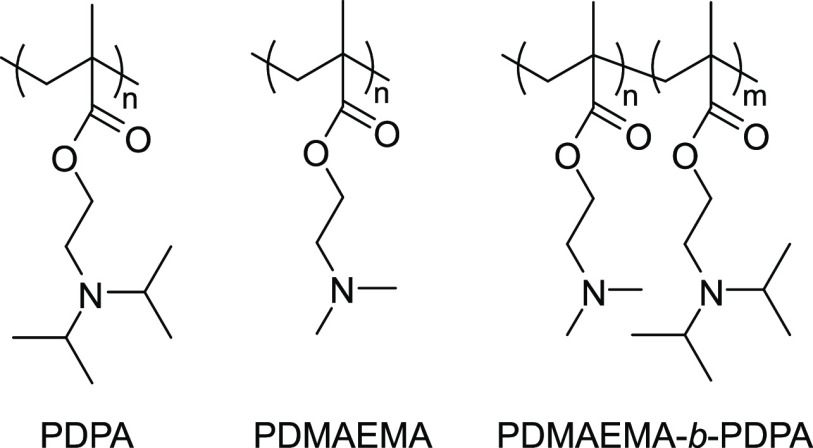
Structures of Poly(diisopropylaminoethyl methacrylate) (PDPA), Poly(dimethylaminoethyl methacrylate) (PDMAEMA), and PDMAEMA-*b*-PDPA Block Copolymer

The polymers, PDPA, PDMAEMA, and PDMAEMA-*b*-PDPA, were synthesized with the reversible addition–fragmentation chain transfer (RAFT) polymerization method.^46^ 4-Cyano-4-(phenylcarbonothioylthio)pentanoic acid was used as the chain transfer agent (CTA) and azobis(isobutyronitrile) as the initiator (I). The structures of the polymers are illustrated in Chart 1, and the characterizations of the polymers are summarized in Table 1. The polymers are labeled with their number-average degrees of polymerization (DP), e.g., PDPA_316_ has a degree of polymerization of 316. The DPs have been determined by ^1^H NMR.

**Table 1:** Overview on Performed Polymerizations

	[monomer]/[CTA]/[I]	reaction conditions	conversion (%)	*M*_n_^b^ (NMR) (g/mol)	*M*_n_^a^ (SEC) (g/mol)	PDI^a^
PDPA_316_	200:1:0.1	90 °C, 17 h	80.0	67 700	14 400	1.69
PDMAEMA_436_	300:1:0.1	90 °C, 17 h	81.7	68 900	18 000	1.51
PDMAEMA_436_ PDPA_49_(PDMAEMA-*b*-PDPA)	220:1:0.2	90 °C, 17 h	45.1	79 400	22 000	1.62

a Measured with size exclusion chromatography.

b Determined by ^1^H NMR using end group analysis.

High reaction temperatures and long reaction times were used to attain high conversions. The moderate polydispersities (Table 1) are explainable by the long reaction times.^47^ One notable detail is that part of the polymer precipitated out of the solution during the reactions. The increased heterogeneity of the system contributes to the polydisperse products.^48^ The synthesis method was based on a previous study, where similar methodology was successfully used to synthesize PDMAEMA.^49^ Nevertheless, the chosen polymerization method may not be the most suitable technique for synthesizing PDPA, and more monodisperse products could be obtained with alternative methodologies. However, considering that this study focuses on the solution behavior of PDPA and no molecular weight dependency is studied, the distributions are reasonably narrow and the polymers were deemed suitable for this study.

The *M*_n_ values defined with NMR and SEC are somewhat different which may indicate some loss of end groups derived from the RAFT CTA. On the other hand, hydrodynamic radius of PMMA, which was used as a standard, is likely different from those of the synthesized polymers. The NMR-spectra of the polymers are shown in Figure S2.

In addition to the homopolymer PDPA, a block copolymer comprised of PDPA and PDMAEMA was studied. The purpose was to synthesize a block copolymer, which would be soluble in water below the cloud point and below pH 6, and form micelle-like structures at high pH and temperature. The hypothesis was based on the similar responses of the two blocks, which appear in different pH ranges. The block copolymer has been prepared with PDMAEMA_436_ as a macrochain transfer agent. In Table 1, the polymer is denoted as PDMAEMA_436_PDPA_49_ but is otherwise referred to as PDMAEMA-*b*-PDPA. The block copolymer has been prepared with PDMAEMA-blocks much longer than the PDPA-blocks; long PDMAEMA-blocks are expected to be able to fully envelop the collapsed PDPA-block above PDPA’s phase transition temperature and form a stabilizing corona around it. The block copolymer was expected to be protonated and soluble at low pH and temperature. However, when pH, temperature, or both increase, the PDPA block becomes insoluble and micelles with a PDPA core and PDMAEMA corona form. pH-induced micellization of PDMAEMA-PDPA,^23^ PDPA-PDMAEMA-PDPA,^42^ and PDPA-PEGMA (poly(ethylene glycol methyl ether methacrylate))^50^ have already been studied at constant temperature taking only pH-dependence into account. This study completes the picture by also introducing the temperature-dependent behavior for such systems.

### Phase Separation of PDPA upon Heating

This section discusses the phase separation behavior of PDPA homopolymer upon heating. The first part presents the cloud point temperatures of various PDPA solutions. After discussing the cloud points, the second part of this section focuses on the enthalpy changes that occur during the phase separation of PDPA.

#### Cloud Point Temperature of PDPA

First, the phase transitions of PDPA homopolymer were observed by measuring the transmittance of polymer solution as a function of temperature. The scattering of light from the dispersion formed above the cloud point temperature (*T*_cp_) is much stronger than that from a homogeneous solution, which leads to decreased transmittance. The effect of pH on the phase transition was studied in the presence of a constant concentration of various salts. Both buffered and nonbuffered solutions were studied. The buffers were prepared by creating the weak acids from their conjugate bases by reacting them with HCl in situ. All samples were prepared by first adding aqueous salt (citrate, phosphate, sulfate, or NaCl), then acid or base and water, and finally the polymer. A more detailed description of sample preparation is given in the Experimental Section.

##### Citrate and Phosphate Ions

The first transmittance measurements were conducted with 20 mM sodium citrate, while the second solution to be studied was 20 mM disodium monohydrogen phosphate. Both solutions were studied at various values of pH. The measurements were conducted with similar heating and cooling rates, 1 °C/min, in the same temperature-range, 5–90 °C. Compared to the solutions with citrate, the phosphate samples phase separated on a wider temperature-range and the transmittance change was not as profound (see Figure S3 for comparison).

It is important to highlight that no phase transitions were observed in pure water. Previously, the thermoinduced phase transition of PDPA has been studied only in buffered solutions.^36^ Transmittance measurement in pure water was conducted on aqueous PDPA at pH 5.5 maximum. Raising the pH higher resulted in polymer precipitating out of the solution.

In neutral solutions, citrate and phosphate have their respective valences of 3 and 2. However, measurements were performed in the pH range of approximately 4.5–6.5. Two p*K*_a_ values of citric acid (p*K*_a2_ = 4.76 and p*K*_a3_ = 6.40^51^) and one p*K*_a_ value of phosphoric acid (p*K*_a2_ = 7.20^51^) are in the vicinity of the studied pH range. Therefore, it should be noted that the valence of the counterions changes depending on the solution pH. Nonetheless, the valence of citrate is always larger compared to the valence of phosphate in similar values of pH, and thus citrate gives higher ionic strengths than phosphate of the same molar concentration.. To assess whether the sharp and prominent transitions resulted from the higher ionic strength, additional measurements were performed in solutions that contained 250 mM NaCl. Even though the ionic strength of the samples with NaCl was much higher than the ionic strength of the citrate solutions, the changes in transmittance were of the same magnitude or smaller than the transitions were with phosphate. It was therefore concluded that the ionic strength of the solution is not the only factor affecting the width and prominence of the phase separation of PDPA.

##### Note on Ionic Strength

Transmittances were also measured in 500 mM NaCl solution and in 20 mM Na_2_SO_4_-solution. SO_4_^2–^ was used as a model ion, as it is bivalent, but unlike citrate and phosphate, it does not buffer the solution in the studied pH-range. No phase transition for the polymer was observed neither in the sulfate solution nor in 500 mM NaCl. Sulfate solutions were studied at different values of pH in the range of 5.1–5.9. For example, at pH 5.1 sulfate solution did not exhibit phase separation, while almost similar (pH 5.0) citrate buffered solution yielded a phase transition at 67 °C. At the high end of the studied pH range, at pH 5.9, PDPA with sulfate once again did not exhibit heat-induced phase separation, while a similar citrate buffered solution phase separated at 34 °C. PDPA precipitated from sulfate solution at pH 6.0 and above.

The measurements were performed on a pH range of approximately 4.5–6.5. At low pH, no cloud point could be observed below 90 °C, which was the highest temperature used in the measurements. On the other hand, at high pH the polymer precipitated already at room temperature. It should be noted that since the adjustment of pH was done by varying the amounts of NaOH and HCl in the solution, even the solutions without added NaCl contain chloride anions and the concentration of Cl^–^ varies with pH. Also, the polymer stock solution contains HCl and thus introduces extra Cl^–^ into the solutions. The phosphate, sulfate, and NaCl solutions’ Cl^–^ concentration was 30 mM due to the PDPA stock solution. Some citrate solutions’ pH was adjusted with additional HCl on top of the stock solution. Even then the Cl^–^ concentration of the citrate solutions was 35 mM at the highest.

##### Transmittance

**Figure 1:**
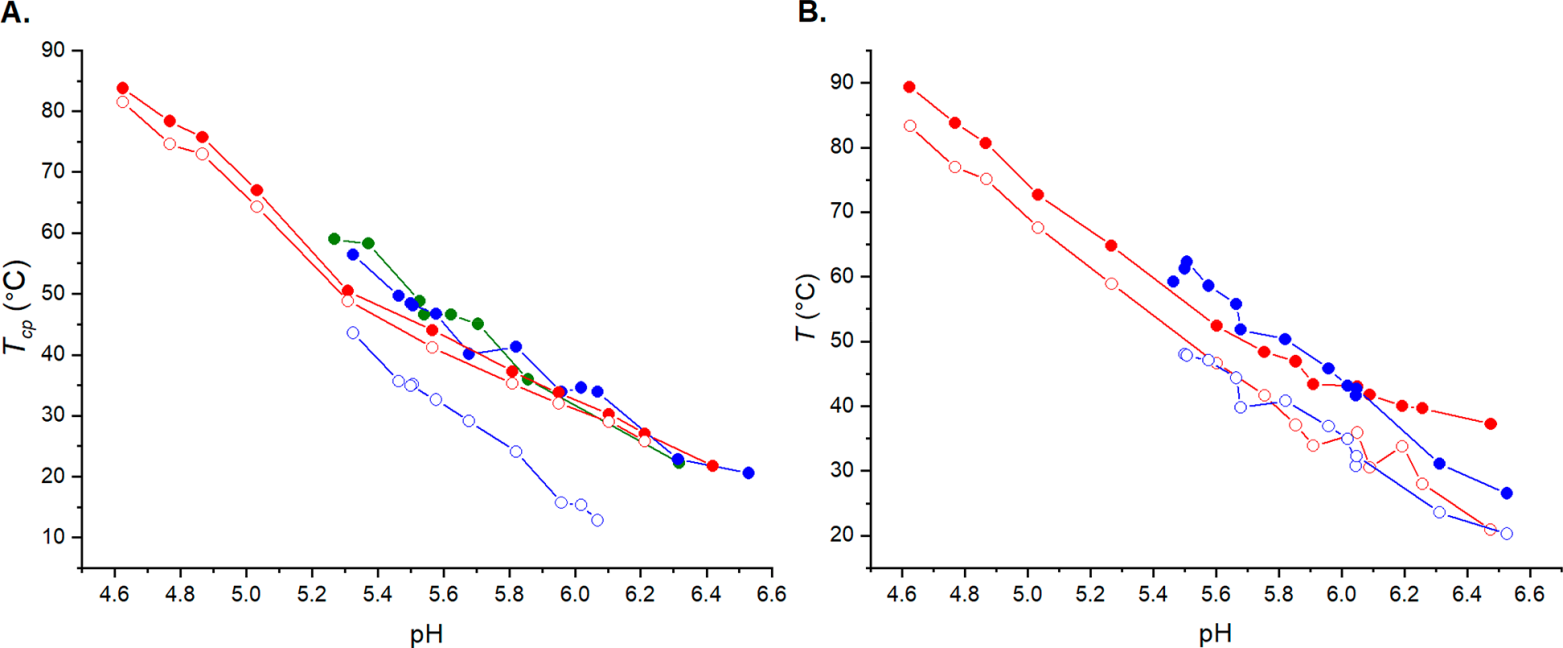
(A) Cloud points (filled symbols) and clearing points (empty symbols) of PDPA with 20 mM sodium citrate (red), 20 mM sodium phosphate (blue), and 250 mM NaCl (green) based on light transmittance measurements. (B) Results of the microDSC measurements conducted for the PDPA solutions. *T*_max_ (filled symbols) and *T*_onset_ (empty symbols) values of citrate solutions (red) and phosphate solutions (blue).

Figure 1A shows the obtained cloud points and clearing points as a function of pH. Clearing point is the point where the sample clears upon cooling. The definitions of the cloud points used in this study have been illustrated in Figure S1. The clearing points could not be determined from the samples in 250 mM NaCl; samples above pH 5.5 were turbid after cooling, and the rest did not exhibit transitions with definable clearing points. The cooling rate was the same as the heating rate, 1 °C/min, which may not have been slow enough for the NaCl solutions. However, it is not known if the solutions clear with time.

##### MicroDSC Measurements

MicroDSC measurements were conducted on aqueous PDPA for the three solutions, which exhibited phase transitions in light transmittance measurements, i.e., 20 mM citrate, 20 mM phosphate, and 250 mM NaCl. The solutions with 250 mM NaCl did not show transitions by means of microDSC. The two buffers, citrate and phosphate, gave differently shaped thermogram curves. The thermograms of the PDPA in the presence of citrate resembled the thermograms of PDMAEMA.^25^ Like in the case of PDMAEMA, the transition starts with a steep increase in the heat capacity and decreases with a gentler slope (Figure S4). Figure 1B depicts the *T*_max_ and *T*_onset_ values of PDPA with citrate and phosphate as a function of pH. The definitions of *T*_max_ and *T*_onset_ are illustrated in Figure S1B.

As mentioned above, microDSC revealed that phase separation of PDPA in 250 mM NaCl could not be observed through calorimetry. However, as seen in Figure 1A, phase separation could be observed through transmittance. This indicates that PDPA employs different mechanisms of the phase separation with different types of counterions. Poly(*N*-isopropylacrylamide) (PNIPAm), for instance, phase separates via separate mechanisms in the presence of kosmotropes and chaotropes.^52^ In the presence of highly hydrated kosmotropes, polarization of water molecules is the main mechanism that affects the phase separation of PNIPAm. Weakly hydrated anions are not able to weaken hydrogen bonding by polarizing water molecules. Instead, they mostly destabilize the hydration of the hydrophobic moieties of the polymer.^52^ It is probable that different anions have different effects on the hydration and consequently the phase separation of PDPA. Verifying this by complementary methodologies, such as molecular dynamics simulations, may be an object of future research.

##### On the Role of pH and Ion Valence

It is also possible that the phase separation of PDPA is related to changes in solution pH. For instance, the phase separation of a copolymer of PNIPAm and poly(4-*N*-2,2,6,6-tetramethylpiperidylacrylamide) has been shown to result in decreased solution pH.^53^ During the phase separation, the ammonium group loses a proton and the lost protons form water with hydroxide anions and lower the solution pH.^53^ Also PDMAEMA exhibits a drop in pH during heating in pure water.^54^ In the case of buffered PDPA, it is likely that the protons lost during the chain collapse are accepted by the buffer anions instead of hydroxide anions, which prevents the change in pH.

NaCl cannot stabilize solution pH as the buffers can. However, heat-induced pH-changes and PDPA’s interdependence of pH and solubility complicate the evaluation of PDPA’s phase behavior in unbuffered solutions. PDPA no longer phase separates upon heating when the concentration of NaCl is increased to 500 mM, which is caused by the fact that at high concentrations, the chloride anions interact with the nonpolar parts of the polymer via weak dispersion forces and cause swelling of the polymer chains.^55−60^ Chloride anions can also bind directly to some of the nonhydrated amine moieties and introduce charges to the chain.^61^ The accumulated ions prevent the formation of interchain aggregates through electrostatic repulsions. Strong interactions between the sulfate anions and PDPA may also explain the lack of cloud points in sulfate solutions. As the anion binds onto the PDPA chains, it causes salting-in behavior.^62−64^ Modeling studies might shed light on the matter in the future.

Figure 1A,B shows that the valence of the counterions has little effect on the phase separation temperature. Cloud point temperatures (*T*_cp_) and *T*_max_ can be determined on a wider pH range for the polymer in citrate solutions than in phosphate solutions. Phase separation upon heating was observed at pH 4.6–6.5 with citrate and at pH 5.3–6.5 with phosphate. Both citrate- and phosphate-containing solutions were turbid at room temperature above pH 6.5. The fact that citrate solution exhibits phase separation at lower pH values than phosphate solution can be rationalized to arise from variations in the buffering ranges. Citrate has a buffering range of 3.0–6.2, while phosphate buffers have a range of pH 5.8–8.0.^51^ Phosphate-buffered PDPA does not phase separate when the buffering capacity is very low, i.e., below pH 5.3. This supports the rationalization that the buffer participates in the phase separation event.

##### Hysteresis and Possible Stepwise Transitions

The width of hysteresis is considerable in the samples with phosphate, and it increases slightly as the pH gets higher (Figure S5). The width of hysteresis was defined as the temperature-difference between cloud points and clearing points. The pH-dependence of the hysteresis suggests that the protonation of the polymer affects the polymer’s solubility upon cooling. When the pH is low and the polymer is protonated, electrostatic repulsions aid the dissolution. Since the dissolution is rapid, hysteresis is small. The phenomenon could not be observed for the transmittance samples with citrate; hysteresis remains small throughout the pH-range. However, according to microDSC, the width of hysteresis increases with pH in both citrate- and phosphate-buffered solutions (see Figure 1B), although the increase in hysteresis was diminutive for citrate buffered PDPA. For phosphate solutions, the increase in hysteresis was comparable to the transmittance measurements. The third p*K*_a_ of citric acid is closer to the studied pH values than the second p*K*_a_ of phosphoric acid, and thus the protonation of the polymer at elevated temperatures assists the polymer dissolution upon cooling.^51^

The variations in hysteresis can be explained through the buffer anions’ effects on the hydration layer. Since citrate has a higher valence than phosphate, it can form a higher number of ion–dipole bonds with water and stabilize the hydration layer and reduce the extent of hydrogen bonding between the polymer and the surrounding water molecules. Therefore, the polymer is less swollen with citrate in the solution than it is with phosphate. Alternatively, ion bridging can explain why the polymer is more swollen in phosphate solution than in citrate solution. The electrolyte that contains condensed, multivalent ions acts as a poor solvent for the polymer chains, which leads to chain collapse.^65,66^ At any rate, citrate, which has a higher valence than phosphate, makes the conformation of the polymer more compact. The larger surface area of PDPA in phosphate facilitates the formation of interchain hydrogen bonds, which stabilize the aggregates. Increased aggregate-stability on the other hand leads to thermal hysteresis. In conclusion, phosphate solution exhibits larger hysteresis because PDPA is more swollen in phosphate solution than in citrate solution.

**Figure 2:**
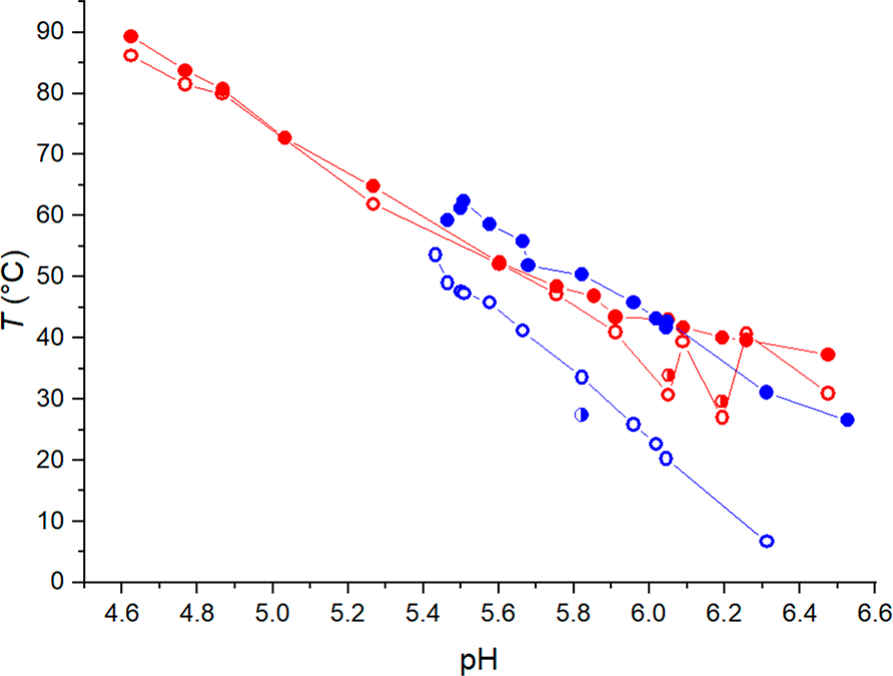
*T*_max_ (filled, red), *T*_m1_ (empty, red), and *T*_m2_ (half-filled, red) of citrate buffered PDPA and *T*_max_ (filled, blue), *T*_m1_ (empty, blue), and *T*_m2_ (half-filled, blue) of phosphate buffered PDPA as a function of pH. The heat capacity of some samples exhibited two minima upon cooling. The minima are depicted as *T*_m1_ and *T*_m2_.

Temperature corresponding to the minimum of the heat capacity (*T*_m_) was determined from the microDSC cooling run thermograms. The cooling rate was 1 °C/min. The obtained *T*_m_ values are presented as a function of pH in Figure 2.

For a few samples at the higher end of the studied pH range, two minima were observed. This suggests that the dissolution of polymer aggregates happens in two stages. PDMAEMA, for example, has displayed similar behavior.^67^ In citrate solution, the thermograms of PDPA exhibited shouldering. This hinted at PDPA undergoing two endothermic steps during heating. However, explicit peak separation was not observed during heating runs. Even though the heating runs did not show separate endothermic events, some of the cooling run thermograms had two peaks. Aggregate dissolution is generally slower than aggregate formation. The appearance of an additional peak in the cooling run suggests that the phase separation (and dissolution) of PDPA occurs in two steps. Examples have been given in the Supporting Information (Figure S6) of thermograms where peak separation occurs and where only peak widening is observed.

In phosphate solution, the thermograms of PDPA were symmetric during the heating run but exhibited shouldering in the cooling run. On one occasion, at pH 5.8, the cooling run thermogram exhibited two discernible peaks. Figure S7 shows thermograms of phosphate buffered PDPA at pH 5.8 and at pH 6.0.

On this account, it is possible that different parts of PDPA dissolve (collapse) separately upon cooling (heating) depending on the surrounding water networks. PNIPAm, for instance, phase separates in two steps in a high concentration of kosmotropic anions. A sufficient concentration of salt weakens the hydrogen bonds to the amide group to the extent that the amide group dehydrates separately from the rest of the molecule.^52^ During the latter step, the hydrophobic hydration water is lost. In addition to PNIPAm, poly(vinyl methyl ether) (PVME) and poly(2-(*N*-morpholino)ethyl methacrylate) (PMEMA) have undergone multistep phase transitions. The first step of the phase separation of PVME is the dehydration of the main chain, which is followed by side chain dehydration.^68,69^ Also for PMEMA, the breakage of hydrophobic hydration and the breakage of hydrogen bonds are seen as two separate steps.^70^

For PDPA, the peak separation was observed at the higher end of the studied pH range and the phenomenon is more prominent in citrate than in phosphate. Considering that the second peak is most pronounced at relatively high pH and with the most highly hydrated anion (i.e., with relatively dehydrated polymer); therefore, the extra peak relates to hydrophobic hydration.

##### Fluorescence Measurements

So far, the phase separation of PDPA has been discussed based on light transmittance measurements and microcalorimetric measurements. The phase separation of PDPA was also studied by fluorescence. Pyrene, a very hydrophobic molecule with low solubility in water, was used as a probe.^71^ The measurements were conducted for polymer solutions with the same three salts that were studied using transmittance measurements (see Figure 1A) with the difference that 1 mL of the water added in the sample was replaced with saturated pyrene solution. The rest of the added water was pure water. The fluorescence properties of hydrophobic probes change above the cloud points.^72^ For instance, neutral LCST-type polymer PNIPAm may incorporate hydrophobic small molecular weight molecules in its collapsed state and change the polarity of the microenvironment surrounding the pyrene.^36^ The ratio of bands I1 and I3 of pyrene fluorescence emission spectrum is sensitive to the solvent polarity. The more polar the environment surrounding pyrene is, the more dominant I1 is over I3.^73^

The I1/I3 ratio of each emission spectrum was calculated and plotted as a function of temperature. This plot for citrate buffered PDPA at pH 5.7 is given as an example in Figure S8. At 15 °C, the I1/I3 is close to 1.8, a typical value for water.^74^ Upon heating to above 40 °C, the ratio decreases rapidly and is only 0.96 at 80 °C. It is close to the I1/I3-value reported for xylene (0.95^74^), meaning that the local polarity of the environment surrounding pyrene has decreased upon heating. On this basis, it may be inferred that PDPA takes up pyrene during phase separation and changes the polarity of pyrene’s microenvironment, thus changing the I1/I3 ratio.

Similar phase transition temperatures were observed by means of transmittance and fluorescence measurements for PDPA solutions with citrate and phosphate. NaCl solutions did not exhibit transitions, i.e., the local polarity near pyrene did not decrease significantly upon heating. This suggests that only a few hydrogen bonds break during the transition, and water content in the aggregates remains high. This explains why no transition was observed via microcalorimetry since evidently the amount of breaking hydrogen bonds is much lower in NaCl solution than in citrate or phosphate solutions. As the enthalpy of transition mostly arises from the changes in hydrogen bonding, the fluorescence results are in line with calorimetric observations that will be discussed in the following section.^75^ The I1/I3 values for PDPA in the NaCl solution for the studied temperatures were very close to the I1/I3 values of pyrene in water (Figure S9). This also indicates that PDPA does not form sites of decreased polarity upon heating in the presence of NaCl. The fluorescence measurements support the suggestion that the PDPA phase separates via separate mechanisms in the presence of buffers and NaCl.

#### Enthalpy Change of the Phase Separation of PDPA

**Figure 3:**
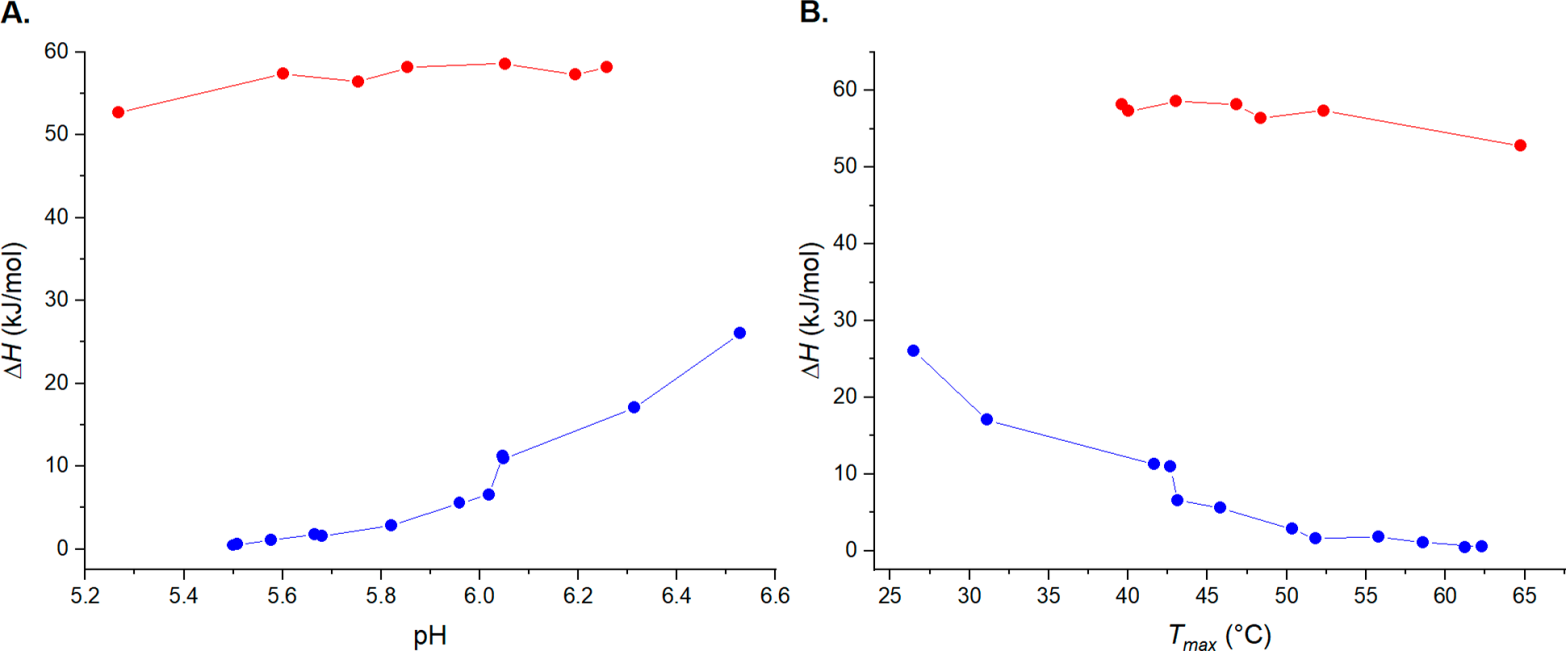
(A) Enthalpy change (Δ*H*) accounted for the phase separation of PDPA in citrate (red) and phosphate (blue) buffers as a function of pH. (B) Enthalpy change (Δ*H*) of the phase transition as a function of *T*_max_ in citrate (red) and phosphate (blue). The enthalpy values are normalized to the concentration of DPA repeating units.

The enthalpy change (Δ*H*) of the phase separation of PDPA was determined from microDSC thermograms. The enthalpy change of PDPA as a function of pH behaves oppositely to PDMAEMA. For PDMAEMA, Δ*H* decreases as the pH increases.^49,67^ In contrast, for PDPA, Δ*H* increases with increasing the pH in both citrate- and phosphate-solutions (Figure 3A).

The slope of the pH dependence of Δ*H* is dependent on the counterion, and the increase is smaller with citrate. The hydrophobicity of PDPA increases with increasing pH, meaning that the number of water–water hydrogen bonds in the hydration layer around the polymer increases as well. Since Δ*H* largely arises from breakage of water–water hydrogen bonds in the water cage that surrounds the hydrophobic moieties, an increased amount of structured water in the hydration layer is seen as increased Δ*H*.^75,76^ The increase in Δ*H* is very slight in the presence of citrate. This can be ascribed to the anion’s effects on the hydration of PDPA. In citrate solution, PDPA is fairly hydrophobic and has a highly structured water cage already at low pH. Further increases in pH cause only relatively small changes in the hydration layer.

Therefore, citrate buffered PDPA only exhibits small changes in Δ*H* with increasing pH. Phosphate buffered PDPA on the other hand is more hydrated. This means that at low pH the hydration layer of phosphate buffered PDPA is not as structured as the hydration layer of citrate buffered PDPA. Increased pH increases the amount of structured water and thus causes major changes in the polymer’s hydration. The pH-induced increase in the amount of structured water in the hydration layer is observed as an increase in Δ*H*.

The values of Δ*H* were considerably larger in citrate solution than in phosphate solution. The differences can be ascribed to buffering capacities and the anions’ effects on the polymer’s hydration. More detailed discussion follows below.

Dehydration of the polymer chains drives the transition upon further heating, and the buffer anions assist in the process. This is related to the deprotonation of the amine groups during phase separation as the buffer anions are the strongest bases available and can accept protons, thus facilitating the phase separation.^54^ Therefore, buffering capacity affects the phase separation process. As mentioned earlier, citrate and phosphate have buffer ranges of 3.0–6.2 and 5.8–8.0, respectively.^51^ The studied pH-range was 4.5–6.5, meaning that the lower end of this pH-range is out of the buffering range of phosphate. Therefore, at the lower end of the studied pH-range, only citrate has significant buffering capacity, which partially explains the efficacy of citrate.

As the buffers accept protons during phase separation, the protonation enthalpies of the two buffers play a part in the observed differences in Δ*H*. The enthalpy of second protonation of phosphate (HPO_4_^2–^ + H^+^ = H_2_PO_4_^–^, p*K*_a_ 7.20) is −5.1 kJ/mol.^77^ The enthalpy of the first protonation of citrate (L^3–^ + H^+^ = HL^2–^, where L = C_6_H_8_O_7_; p*K*_a_ 6.40) is 3.3 kJ/mol.^78^ The second protonation enthalpy (HL^2–^ + H^+^ = H_2_L^–^, p*K*_a_ 4.76) is −2.0 kJ/mol.^78^ The positivity of the first protonation enthalpy explains the large, endothermic change observed in citrate buffered solutions. The enthalpy change of the second protonation is negative but still lower than the protonation enthalpy of phosphate. The exothermic protonation of phosphate reduces the endotherm, which results in the lower phase transition enthalpies.^76^

##### Dependence of the Enthalpy Change on the Choice of Buffer

The experimental Δ*H* at pH 6.3 were 58 kJ/mol in a citrate solution and 17 kJ/mol in a phosphate solution, meaning that Δ*H* was thrice the value in citrate compared to phosphate. This is the first time that such a strong effect of type of buffer on the thermodynamics of phase transition of a weak polycation has been observed. Previous studies have not taken into account the obvious strong effect of polymer–buffer anion interactions. The strong effect should be discussed in future studies on the matter as well.

Calculating with the Henderson–Hasselbalch equation, 33% of the polymer is protonated at the highest pH where transitions are observed in both buffers at pH 6.3. Since the degree of protonation of a polyelectrolyte can only be estimated from one p*K*_a_, it was calculated using an apparent p*K*_a_ from the literature, 6.0.^23^ Upon the assumption that the polymer deprotonates completely during the phase transition and the buffer anions take up all protons, enthalpy changes of 1.1 kJ/mol and −1.7 kJ/mol for citrate- and phosphate-solutions are obtained. Therefore, the endotherm is 2.8 kJ/mol larger in citrate than in phosphate. This difference in protonation enthalpies is one way to rationalize the variations between different counterions. The system contains various ions and the citrate- and phosphate-ions may not accept all the protons. It is also worth mentioning that the transition enthalpy of almost completely deprotonated PDMAEMA is much lower (1–2 kJ/mol^25,49^) than the Δ*H* of PDPA.

PDPA is a weak polyelectrolyte and thus exhibits complicated protonation behavior. For example, the p*K*_a_ of PDPA is likely to vary depending on the solution and temperature.^79^ Therefore, the changes in p*K*_a_ can affect the Δ*H* of the phase transition. Still, as stated above, the enthalpy changes were only used as a means to estimate the differences between citrate and phosphate buffers.

The differences in Δ*H* mean that the endothermic process related to the phase transition of citrate buffered PDPA is more profound than the process in the phosphate solution. This suggests that more hydrogen bonds break upon the phase separation in citrate solution. Consequently, in addition to the dissimilarities related to buffering capacity, the differences can be explained by the anions’ ability to bind to the hydration layer surrounding the polymer. As discussed above in the context of hysteresis, citrate ions are likely to hydrate more strongly than phosphate anions, as they are able to form a higher number of ion–dipole bonds than phosphate. The highly hydrated citrate anions can polarize water molecules of the polymer’s hydration layer and thus weaken the bonding of the water molecules to PDPA; similar anion-induced weakening of hydrogen bonding has been observed for PNIPAm.^80^ Consequently, the stability of the hydration layer increases. Since the breakage of the hydrogen bonds between water molecules is the main contributor to the enthalpy change upon phase separation, the enthalpy-change is larger in the presence of citrate than in the presence of phosphate.^75,76^

The discussion above leads to the conclusion that the different Δ*H* in citrate and phosphate buffers are a combined result of different buffering capacities and dissimilar effects on the polymer’s hydration layer.

It is also worth noting that solution pH also affects the dissociation degree of the buffer salts. Therefore, the valences of citrate and phosphate undergo changes within the studied pH range of 4.5–6.5. Citric acid, for instance, has p*K*_a_ values of 3.13, 4.76, and 6.40.^51^ Two of these values are within the studied pH range. This suggests that the solution can contain varying ratios of species of different valences at different pH. Phosphate on the other hand has only one p*K*_a_ in the vicinity of the studied pH range (7.20^51^) and exhibits hence less variation in the salt valence.

Figure 3B shows the Δ*H* accounted for the phase transitions as a function of *T*_max_. As is typical for other polymers that phase separate upon heating but opposite to PDMAEMA, the Δ*H* of the phase transition decreases with increasing *T*_max_.^75,81^ The breakage of the water–water hydrogen bonds surrounding the polymer’s hydrophobic parts contributes to the enthalpy of the phase transition. The polar groups of the polymer form hydrogen bonds with surrounding water molecules below the phase separation temperature. Nonpolar groups on the other hand are encapsulated in a hydration layer consisting of surrounding water molecules. When the cloud point is surpassed, the hydration layer’s water–water hydrogen bonds break. The phase separation temperature is greatly determined by the hydrophilicity–hydrophobicity balance of the polymer. The addition of hydrophilic moieties often increases the phase transition temperature of LCST-type polymers.^75,82−84^ If the phase separation temperature is high, the polymer is probably rather hydrophilic and the water–water hydrogen bonds in the hydration layer are relatively few.^76^ Therefore, Δ*H* decreases with increasing *T*_max_.

##### PDPA vs PDMAEMA

As mentioned earlier, PDPA and PDMAEMA exhibit different phase separation thermodynamics, namely, PDPA gives larger values of Δ*H*, and PDPA and PDMAEMA exhibit opposing Δ*H* versus pH and Δ*H* versus *T*_max_ behaviors. The Δ*H* as a function of *T*_max_ behavior of PDPA can be observed also upon cooling. That is, the Δ*H* of the polymer’s dissolution upon cooling decreases with increasing *T*_m_ (Figure S10).

The Δ*H* of PDPA were higher than the Δ*H* of PDMAEMA generally is. This can be attributed to differences in the two polymers’ hydrophilicities and phase separation mechanisms. Previous experimental and theoretical studies have shown that the collapse of PDMAEMA is governed by its carbonyl group, while the collapse of PDPA is defined by the hydrophobic interactions of the diisopropylaminoethyl group.^26,36^ PDMAEMA is highly hydrated even in its globular conformation. This is because the PDMAEMA phase separates as a result of partial dehydration of the carbonyl group. The relatively polar dimethylaminoethyl group on the other hand promotes water uptake of the polymer. This means that PDMAEMA transforms from a coiled formation into a rather highly hydrated globule. In comparison, PDPA is in a globule-like conformation already in its soluble state and adopts an even more compact conformation as the diisopropylaminoethyl group dehydrates.^26,36^ Since Δ*H* largely arises from the breakage of hydrophobic hydration, it is reasonable to conclude that the variations in the polymers’ hydrophilicities were the reason behind the differences in Δ*H* of PDPA and PDMAEMA.^75,76^ PDPA, which has stronger hydrophobic hydration, exhibits larger values of Δ*H*.

The second difference concerns the development of Δ*H* with pH and *T*_max_. At low temperatures, PDMAEMA, unlike PDPA, is well in contact with water at all pH values. PDMAEMA is thus well hydrated even when the monomer units are noncharged.^36^ This behavior contrasts PDPA, which loses contact with water with increasing pH at low temperatures.^36^ Increased hydrophobicity of PDPA is accompanied by an increase in the number of water–water hydrogen bonds in the hydration layer around the polymer. The breakage of these hydrogen bonds is seen as a change in enthalpy. Since PDMAEMA does not become hydrophobic with increasing pH, the number of hydrogen bonds in the hydration layer does not increase with pH. Instead, Δ*H* of PDMAEMA is highly dependent on the extent of the amine group’s protonation. The more protonated PDMAEMA is (the lower the pH is), the larger is the number and strength of the hydrogen bonds between the polymer and water. Therefore, the phase transition is more endothermic and occurs at higher temperatures at low pH.^85^ In summary, Δ*H* of PDPA is defined by the hydrophobic hydration of the polymer, while the Δ*H* of PDMAEMA is defined by the extent of its hydrogen bonding with water.

### Phase Separation Behavior of PDMAEMA-*b*-PDPA Block Copolymer and PDMAEMA Homopolymer

PDPA is insoluble at room temperature above pH 6.5. PDMAEMA on the other hand can be soluble even at pH 10 and phase separates upon heating.^49^ Therefore, a block copolymer consisting of PDMAEMA and PDPA is expected to form micelle-like structures with a PDPA core and a PDMAEMA corona at high pH or temperature.

The block copolymer was studied at three pH values (6, 8, and 10) and PDMAEMA homopolymer at two (pH 8 and 10). In addition to pH 6 where the PDPA homopolymer phase separates upon heating, the higher pH values of 8 and 10 were studied, because PDMAEMA, which makes up the majority of the block copolymer, exhibits phase separation upon heating in a pH range of 7–10.^49^

**Figure 4:**
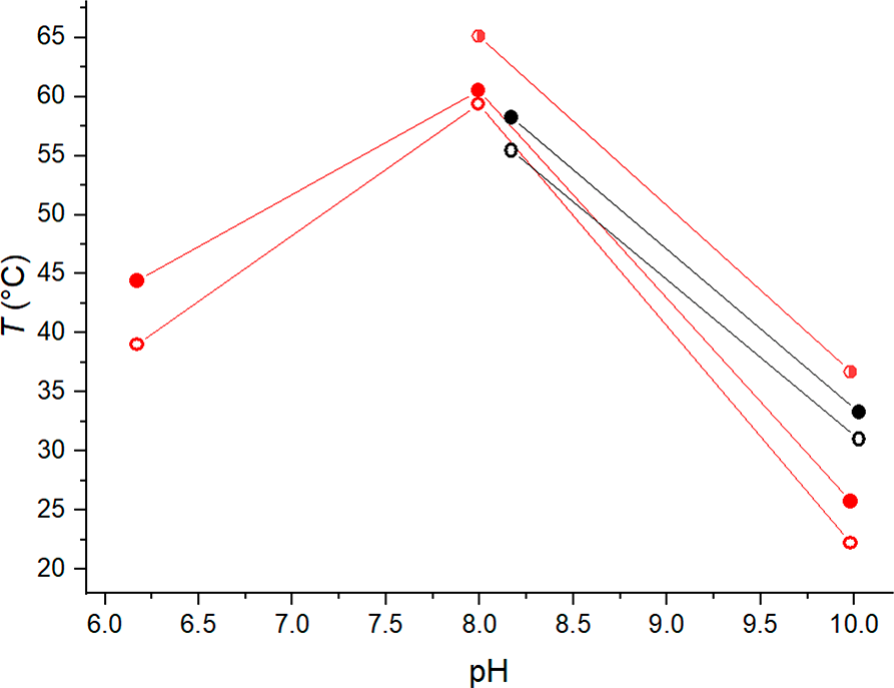
*T*_max_ of PDMAEMA (black, filled symbols) and PDMAEMA-*b*-PDPA (red, filled and half-filled symbols), and *T*_onset_ of PDMAEMA (black, open symbols) and PDMAEMA-*b*-PDPA (red, empty symbols) as a function of pH.

*T*_max_, *T*_onset_, and the enthalpy changes were determined from the thermograms. The obtained *T*_max_ and *T*_onset_ values as a function of pH are shown in Figure 4. The block copolymer displayed a phase transition only when the solution was buffered. Unbuffered PDMAEMA-*b*-PDPA did not undergo phase separation at pH 6. Measurements at pH 8 and 10 were conducted only in buffered solutions. The pH 6 and 8 samples were buffered with phosphate. pH 10 samples were buffered with borate, since the buffering capacity of phosphate is very low at pH 10.

Figure 4 shows that the phase separation temperature of the block copolymer is the highest at pH 8. The phase separation temperature of PDMAEMA-*b*-PDPA at pH 6 was a close match to the phase separation temperature of PDPA homopolymer at similar pH. In contrast, at pH 8 and 10, the phase separation temperature of the block copolymer resembled that of PDMAEMA homopolymer. However, the addition of a hydrophobic block (PDPA) slightly lowered the phase separation temperature compared to PDMAEMA. This attests to the fact that the phase behavior of the block copolymer was defined by the PDPA block at pH 6, while at higher pH PDMAEMA was the defining block. For this reason, the Δ*H* of the block copolymer was normalized to PDPA at pH 6 and to PDMAEMA at pH 8 and 10 (Figure S11).

The block copolymer exhibited two-step transitions at pH values of 8 and 10 (Figure S12). PDPA is almost completely deprotonated at pH 8 and 10 and is thus collapsed already at room temperature. Therefore, below the phase separation temperature, at pH 8 and 10, PDMAEMA-*b*-PDPA forms micelles in which collapsed PDPA forms a core that is surrounded by a hydrated PDMAEMA block. The two-step transitions at pH 8 and 10 suggest that different parts of the block copolymer collapse independently. Two-step transitions have been observed for particle-bound PNIPAm brushes, dendritic micelles, and hydrophobically modified PNIPAm, for instance. In these cases, the phenomenon was ascribed to the presence of a two-layered polymer shell.^86−89^ Also PDMAEMA-*b*-PDPA might have self-assembled into micelles with a two-layered shell. The shell contains a layer that is in close proximity to the PDPA core and a layer that is exposed to water. The inner layer of the shell is partially dehydrated due to hydrophobic effects of the PDPA core.^88^ Hence, the lower temperature transition arises from the collapse of the inner layer of the micelle corona. The higher temperature transition on the other hand can be attributed to the collapse of the highly hydrated outer layer of the corona.

The differences between the homopolymer and the block copolymer are more pronounced if enthalpies of the transitions are compared. The enthalpy of PDMAEMA-*b*-PDPA increases as a function of pH; the same is observed for PDPA (Figure S11). As is discussed above, for PDPA the enthalpy of the phase transition increases with pH, which is opposite to PDMAEMA. The DMAEMA content of the polymer is larger than that of DPA, which is why one might expect the behavior of PDMAEMA-*b*-PDPA to be akin to PDMAEMA. As discussed above, the phase separation of PDMAEMA is defined by the polymer’s extent of hydrogen bonding with water. PDPA on the other hand is more hydrophobic and thus its behavior is more dependent on the properties of the water cage that surrounds the polymer. The addition of a hydrophobic block (i.e., PDPA) to PDMAEMA may have caused the polymer to behave more like PDPA. That is, Δ*H* of the phase separation of PDMAEMA-*b*-PDPA increases as pH and strength of the water cage increase. However, identifying the reasons for why the Δ*H* development of PDMAEMA-*b*-PDPA resembles that of PDPA requires further research.

### Size of the Multimolecular Aggregates of PDPA, PDMAEMA, and PDMAEMA-*b*-PDPA Block Copolymer

PDPA, PDMAEMA, and PDMAEMA-*b*-PDPA solutions were studied by means of light scattering measurements at different temperatures. PDPA was investigated in citrate and phosphate buffers around pH 6. PDMAEMA was studied at pH 8 and 10, and the block copolymer at pH 6, 8, and 10; all of them buffered using phosphate for pH 6 and 8 and borate for pH 10. The maxima of the obtained hydrodynamic diameter distributions were plotted as a function of temperature. For example, such a plot for citrate buffered PDPA at pH 5.6 is shown in Figure S13 in the Supporting Information. The graph shows that the size of the particles starts to increase around 45 °C, goes through the maximum at around 50 °C, and settles down to a constant value. This derives from competition between inter- and intrachain interactions. The polymer chains collapse until the point where intra- and interchain repulsions stop the collapse and stabilize the aggregates.^90^ When the solution is cooled down, the aggregates swell before dissolution; PNIPAm exhibits similar behavior.^90^

**Figure 5:**
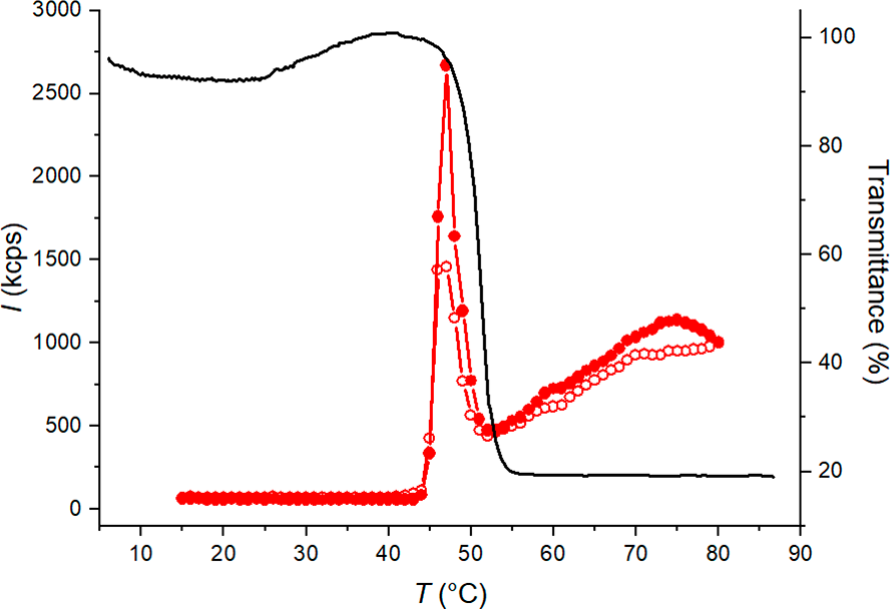
Scattering intensities (*I*) of heating (red filled symbol) and cooling (red empty symbol) of PDPA in 20 mM citrate solution at pH 5.6. In addition, the transmittance as a function of temperature of a similar sample has been shown as a black line.

The polymer aggregation was also monitored using the intensity of the scattered light (*I*) (Figure 5). When the scattering intensity of the same sample was monitored as a function of temperature, the same behavior was observed as with the particle sizes at different temperatures; a large aggregate formed at around 45 °C and shrunk upon further heating. In addition, the same swelling before dissolution was observed in the cooling run. When the scattering intensity is compared to the transmittance curve of a similar sample, it is seen that the scattering intensity starts to increase before transmittance starts to decrease. Light scattering detects aggregate formation already before the solution becomes opaque and then cloudy. Transmittance drop on the other hand is only observed once macroscopic precipitation occurs. Therefore, dynamic light scattering (DLS) gives a lower phase transition temperature than transmittance does. However, another factor to take in account is the fact that the transmittance and DLS measurements were conducted with different heating rates.

DLS measurements were measured stepwise upon temperature change, and the average heating rate was only one-third of the heating rate in transmittance measurements. Lower heating rates result in lower transition temperatures. MicroDSC, which was measured with the same heating rate as the transmittance measurements, shows that citrate buffered PDPA at pH 5.6 starts to phase separate at 47 °C. According to DLS (Figure 5 and Figure S13), the phase separation starts already at 45 °C. Evidently, the lower heating rate gave slightly lower phase transition temperatures. Still, it should be noted that the phase transition temperatures obtained with DLS were not generally compared to the phase transition temperatures obtained through other methods. The purpose of Figure 5 is merely to show that the aggregation temperature reasonably agrees with the transmittance drop related to the phase transition of PDPA.

**Figure 6:**
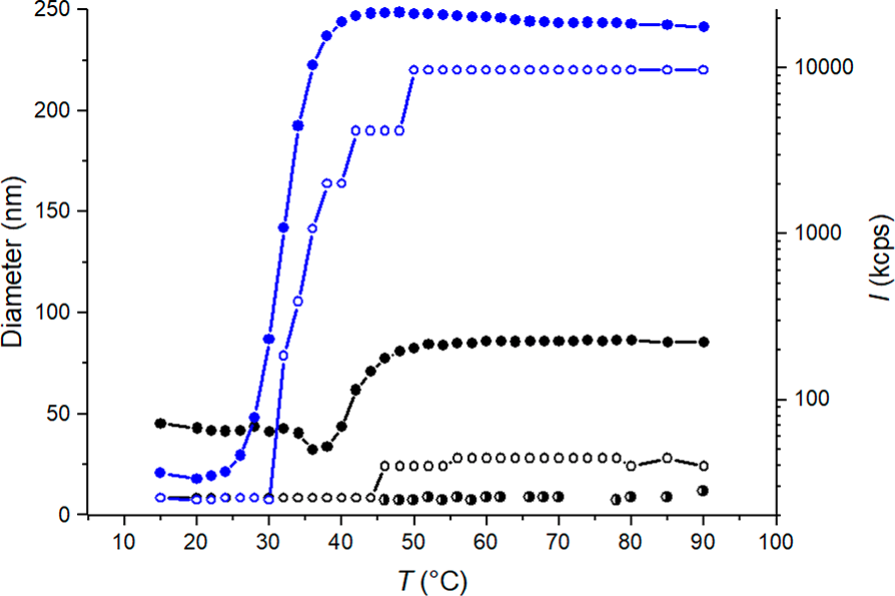
Scattering intensity (filled symbol) and intensity-average diameter (empty symbol) of PDPA with 20 mM phosphate at pH 6.0 (blue). The scattering intensity (filled symbol, black) and the average particle sizes of PDMAEMA-*b*-PDPA at pH 6.2. The measuring points, in which the intensity-average size distributions of the block copolymer had two maxima and are depicted with two black symbols: empty and half-filled. The lines are a guide for the eye.

As for the differences in the phase transitions of PDPA in different buffers, light scattering support observations obtained using microDSC and transmittance measurements: the transitions were narrower for the citrate solutions than they were for phosphate solutions. For light scattering measurements, the width of transition was defined as the temperature difference between the point where the aggregates started to form and the point where the size of the aggregates is stabilized. Citrate also yielded larger aggregates, which shrunk quickly. In a phosphate solution, the size of the aggregates remained at a constant size all the way to the end of the heating run. Figure 6 compares the sizes of the multimolecular aggregates formed above the phase separation temperature and the scattering intensities of PDPA at pH 6.0 and PDMAEMA-*b*-PDPA at pH 6.2. The comparison shows a large size difference between the formed aggregates. The block copolymer forms smaller aggregates. Below 30 °C, the individual PDMAEMA-*b*-PDPA chains are well swollen. Above the cloud point temperature of PDPA, the PDPA block collapses. However, the PDMAEMA block remains in a soluble, extended conformation and forms a stabilizing corona around the aggregated PDPA core. One can expect that because at pH 6, the charged PDMAEMA remains soluble throughout the studied temperature range. The presence of a hydrophilic component in the block copolymer resulted in an increase in the phase transition temperature compared to PDPA. This is a typical feature of thermosensitive polymers, which has been observed, e.g., for PNIPAm.^75,76^ The aggregation of PDPA homopolymer starts around 30 °C, whereas the onset occurs only after 40 °C for the block copolymer.

At pH 8 at room temperature, PDPA is insoluble. Therefore, the block copolymer forms micelle-like structures with almost completely dehydrated PDPA in the core. As discussed above, the micelles have two-layered PDMAEMA shells. Gradual collapse of the shell occurs as the solution is heated (Figures S14 and S15).

Also, at pH 10, PDPA is almost completely dehydrated before heating. The collapse of the micelle shell starts already in the early stage of the heating process. Figure S16 compares the scattering intensities of PDMAEMA and PDMAEMA-*b*-PDPA as a function of temperature. Comparison of the average particle sizes of PDMAEMA and PDMAEMA-*b*-PDPA shows that PDMAEMA aggregates at a slightly higher temperature than the block copolymer does. The addition of a hydrophobic component (i.e., PDPA) decreases the phase separation temperature of the polymer. PDMAEMA-*b*-PDPA displays two maxima in the scattered intensity, one at 55 °C and another at 70 °C. The first maximum was ascribed to the collapse of individual micelles and the second maximum to micelle associations.

Since PDPA is not soluble in neutral and basic water solutions, PDMAEMA-*b*-PDPA forms micelle-like structures in neutral and basic solutions below *T*_cp_. PDPA forms the nonhydrated core of the micelles, while PDMAEMA remains soluble and forms the corona.^23^ At pH 6 at 15 °C, the average diameter of the block copolymer was 9 nm, whereas at pH 8 the average diameter was almost 16 nm. A further increase in pH does not provoke significant changes in the polymer diameter; the average diameter was 18 nm at pH 10. At pH 6, PDMAEMA-*b*-PDPA is molecularly dissolved. At higher pH values, the hydrophobic interactions overcome the electrostatic repulsions in the PDPA block and micelles are formed. A diblock copolymer with PDPA and PEGMA blocks exhibits similar behavior.^50^ The formed micelles are quite small. The micelle diameter evidently only doubles compared to the unimers. The diameter of the DMAEMA-DPA micelles has been observed to increase with increasing DPA content.^23^ Since the studied block copolymer contains only approximately 10% DPA, the resulting micelles are small. It may be assumed that the low aggregation number results from the low core block length. The high DMAEMA content results in relatively hydrophilic micelle surfaces with a low driving force for aggregate growth.

As discussed earlier, the properties of PDPA homopolymer are strongly dependent on the added anions. Therefore, the micelles are likely to be influenced by anions as well. Hence the anions’ effects on micelle morphology, micelle hydration, aggregation number, etc. ought to be explored in future contributions.

## Conclusions

This contribution sheds light on the phase behavior of an underrepresented dual stimuli-responsive polymer PDPA. Homo- and block copolymers of DPA were synthesized successfully. This study shows that the phase separation behavior of PDPA depends on the type of added salt or ionic strength or both. Furthermore, the solution behavior is dependent on the pH of the solution, i.e., the polymer’s degree of charging. To the best of the authors’ knowledge, this is the first time when the effect of buffering on phase transitions of PDPA was studied.

The main finding of this study was that the phase separation of PDPA upon heating exhibits strong counterion dependency. In fact, phase separation can only be observed in the presence of salts; transitions were not observed in water. The effect of citrate-, phosphate-, chloride-, and sulfate-salt additions was studied. PDPA underwent phase separation upon heating in citrate-, phosphate-, and NaCl-solutions. No transition could be observed in sodium sulfate. This is ascribed to strong interactions between the anion and the polycation.

PDPA had similar phase transition temperatures in citrate and phosphate buffers. However, the presence of citrate led to more pronounced and narrow transitions compared to the phosphate-containing solutions. Furthermore, the transitions could be determined in different pH ranges, and the buffers affected the thermodynamics of the phase separation.

The dissimilar pH ranges in which phase separation was observed were attributed to the buffering ranges of citrate and phosphate. Phosphate, whose buffering range only covered a part of the studied pH values, gave phase transitions in a narrower pH range than citrate, which is able to buffer the entirety of the investigated pH range.

The differences between citrate and phosphate buffered PDPA were evident in the thermodynamics of the phase separation. The values of Δ*H* were considerably larger in citrate solution than in phosphate solution. This is a combined effect of variations in buffering capacity, buffer anions’ protonation enthalpies, and buffer anions’ effects on the structure of PDPA’s hydration layer. In phosphate solution, the hydration layer of PDPA is considerably less structured than in citrate. This leads to a loss of a smaller amount of water in the former compared to the latter.

The phase separation of PDPA was studied in highly concentrated NaCl solutions in order to study the effect of the ionic strength. Even though the NaCl solutions had the highest ionic strength, their phase separations were even less pronounced and occurred in wider temperature-ranges than the phase transitions of PDPA with phosphate. Thus, it was concluded that the ionic strength was not the only reason behind the differences between the anions’ effects on the phase separation behavior of PDPA. The phase transition of PDPA in 250 mM NaCl was observed by means of transmittance measurements but could not be observed using microcalorimetry nor fluorescence measurements. This was attributed to PDPA undergoing phase separation via separate mechanisms in the presence of buffers and NaCl. The difference mainly arises from the fact that in NaCl solution, PDPA does not lose as much water molecules as in the buffers. This leads to a lesser amount of hydrogen bonds broken (low phase transition enthalpies, not detectable by DSC) and more hydrated associates (which cause no detectable changes in pyrene fluorescence spectrum).

The phase separation of PDMAEMA is molar mass dependent,^85^ and thus the effect of the molar mass on the phase transition temperature of PDPA ought to be studied in the future.

Since PDMAEMA and PDPA are both pH- and thermally responsive but phase separate within different pH-ranges, the solution behavior of a block copolymer consisting of PDMAEMA and PDPA blocks was studied. PDMAEMA-*b*-PDPA formed micelles as a response to pH and temperature changes, where DMAEMA formed a solvated micelle corona and DPA a hydrophobic core. The transition temperature was affected by the other block; compared to PDPA, the transition temperature of the block copolymer was higher due to the increased hydrophilicity from PDMAEMA. Comparison with PDMAEMA on the other hand shows that the addition of a more hydrophobic PDPA block decreases the transition temperature of the polymer.

## Supporting Information Available

^1^H NMR spectra of the polymers, exemplifying transmittance and DSC curves, and additional data from DSC, fluorescence, and DLS measurements (PDF)
