# Supplementary material for: Phase Separation of Aqueous Poly(diisopropylaminoethyl methacrylate) upon Heating
Source: Langmuir. 2021 Nov 9;38(17):5135–48. doi: 10.1021/acs.langmuir.1c02224 (PMC9069861; doi:10.1021/acs.langmuir.1c02224)
Supplement: Supplementary file 1 — la1c02224_si_001.pdf [file la1c02224_si_001.pdf]

Supporting Information: Phase Separation of Aqueous Poly(diisopropylaminoethyl methacrylate) upon Heating

Linda Salminen[a], Erno Karjalainen[b]*, Vladimir Aseyev[a]*, Heikki Tenhu[a]

[a]Department of Chemistry, University of Helsinki, P.O. Box 55 (A.I. Virtasen aukio 1), FIN-00014 HY Helsinki, Finland
[b]VTT Technical Research Centre of Finland Ltd, P.O. Box 1000, FI-02044 VTT, Espoo, Finland

* corresponding authors, E-mail: erno.karjalainen@alumni.helsinki.fi (E.K.) and vladimir.aseyev@helsinki.fi (V.A.).

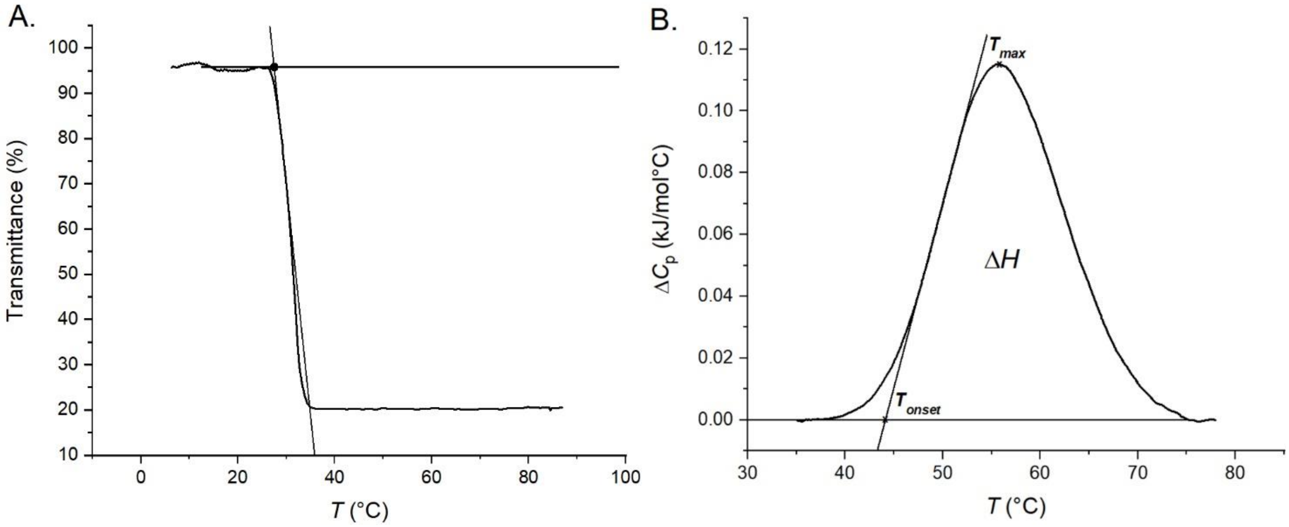

Figure S1. Illustrations of the definitions used in this study: A. Cloud points and clearing points are determined as the intersection of two tangents. B. Microcalorimetric endotherm, where ΔH is the area under the curve and T_{max} the peak value. T_{onset} is determined as the intersection of the two tangents.

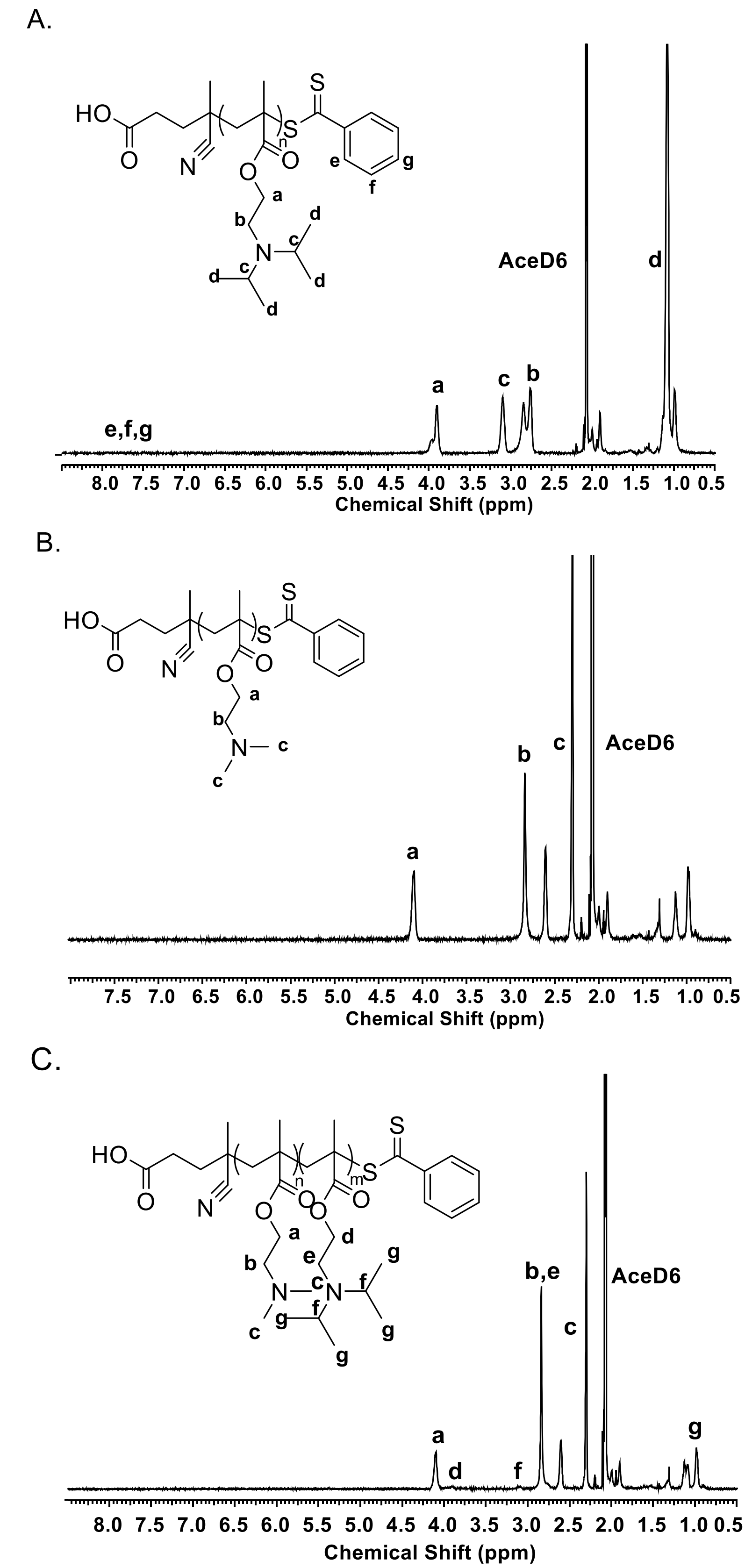

Figure S2. NMR-spectra of A. PDPA, B. PDMAEMA, and C. PDMAEMA-*b*-PDPA. All of the spectra were measured in deuterated acetone (AceD6).

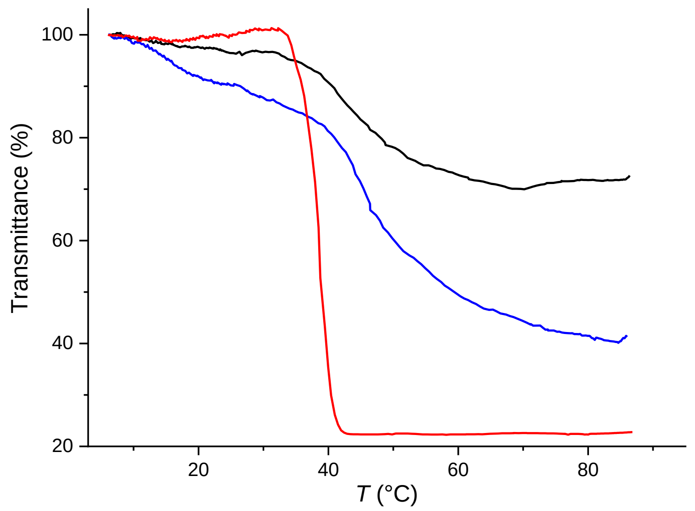

Figure S3. Comparison of transmittance curves of PDPA with different salts. Red: 20 mM citrate, pH 6.0; Blue: 20 mM phosphate, pH 6.0, and Black: 250 mM NaCl, pH 5.9.

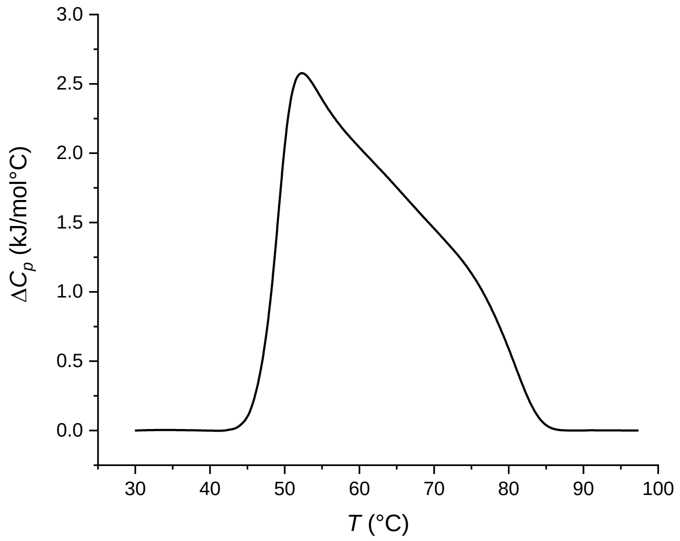

Figure S4. A thermogram of PDPA with 20 mM citrate, pH 5.6. The enthalpy value is normalised to the concentration of DPA repeating units.

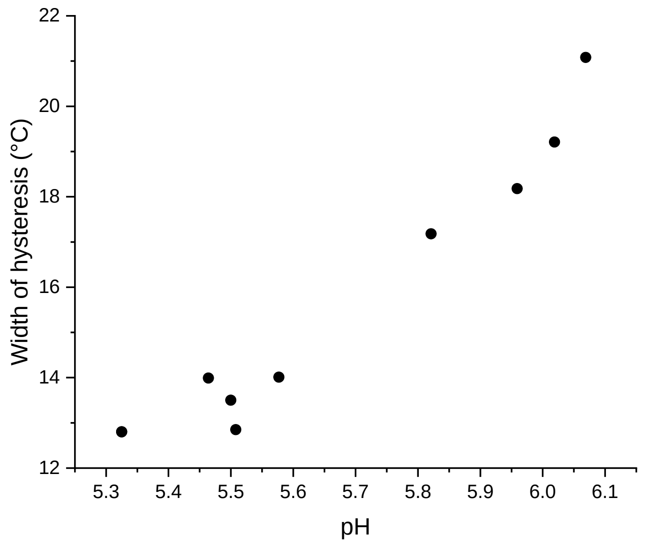

Figure S5. Width of hysteresis of phosphate buffered PDPA as a function of pH. The width of hysteresis was defined as the temperature-difference between the cloud point (T_c) and the clearing point of transmittance measurements.

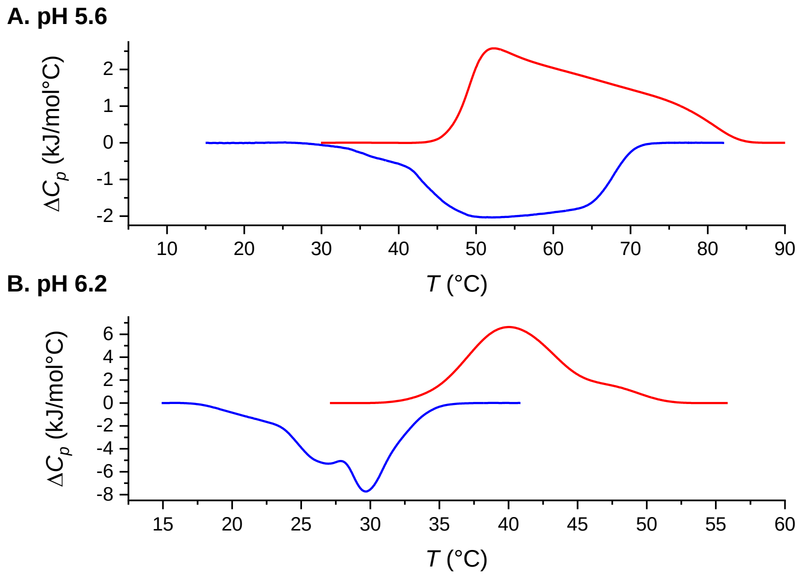

Figure S6. Thermograms of PDPA in 20 mM citrate at pH 5.6 (A), and at pH 6.2 (B). The heating runs have been depicted with red lines and cooling runs with blue lines.

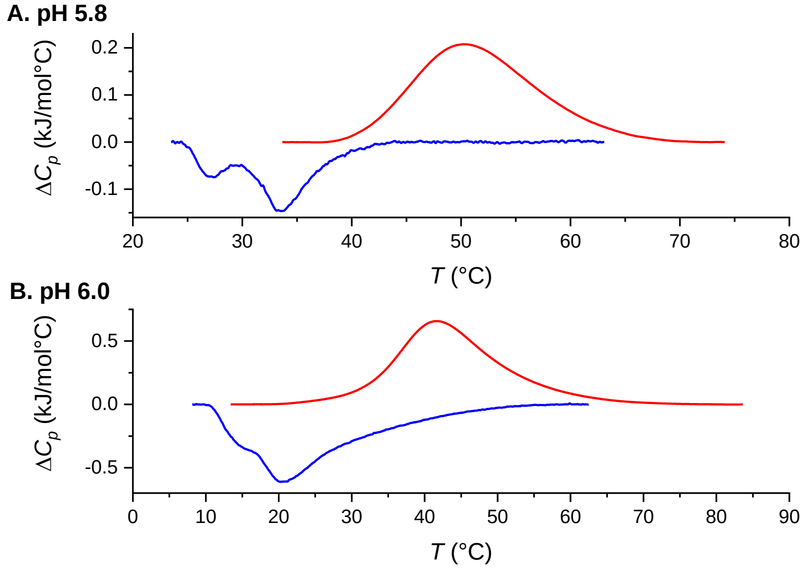

Figure S7. Thermograms of PDPA in 20 mM phosphate at pH 5.8 (A) and at pH 6.0 (B). The heating runs have been depicted in red and cooling runs in blue.

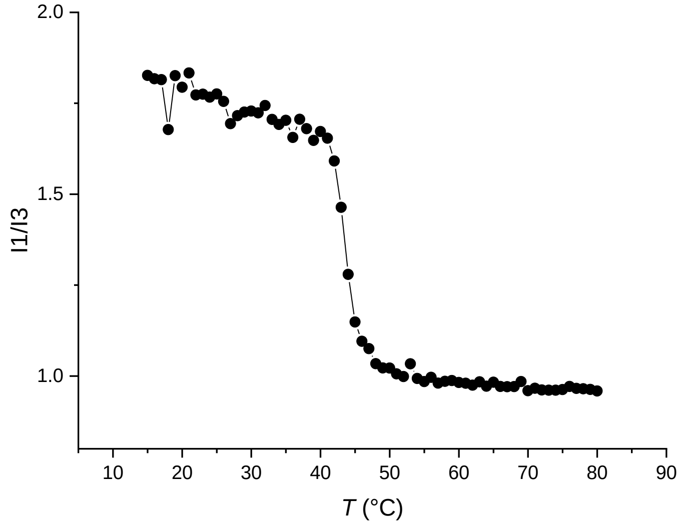

Figure S8. I1/I3 of citrate buffered PDPA (pH 5.7) as a function of temperature. The line is a guide to the eye.

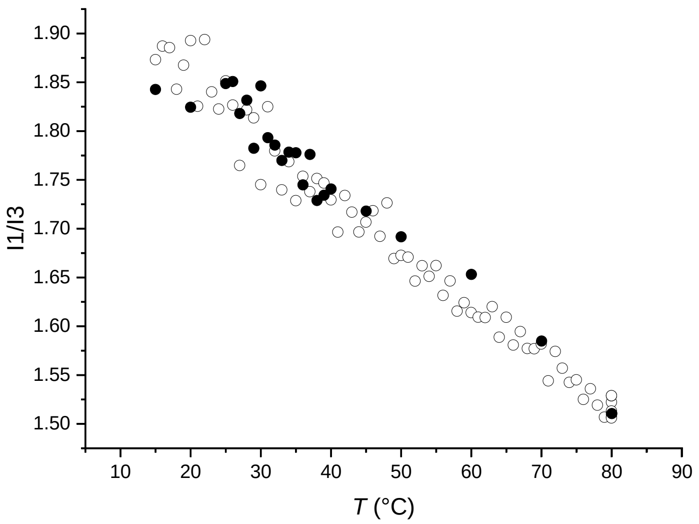

Figure S9. The I1/I3 as a function of temperature for a solution of PDPA with 250 mM NaCl at pH 5.6 (○) and for an aqueous solution of pyrene without any polymer (●).

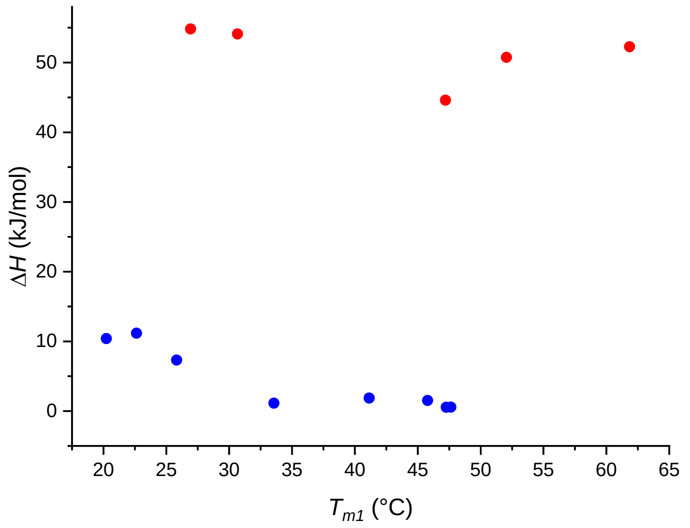

Figure S10. The enthalpy change (ΔH) of the aggregate dissolution as a function of T_{m1} in citrate (red) and phosphate (blue). The enthalpy values are normalised to the concentration of DPA repeating units.

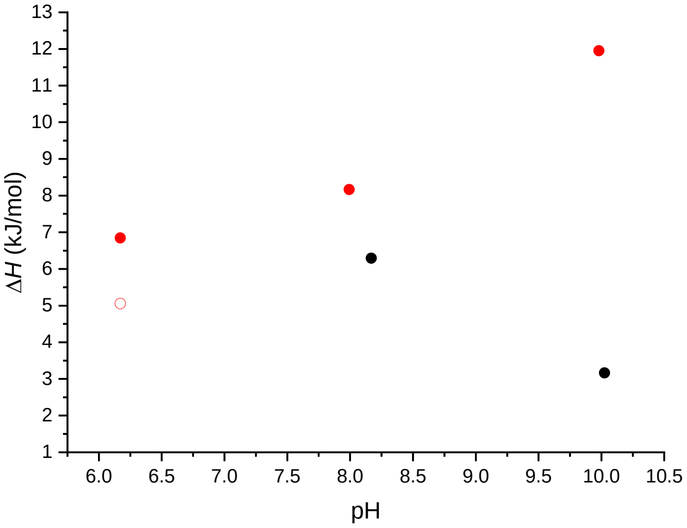

Figure S11. The enthalpy changes of the phase transitions of PDMAEMA-*b*-PDPA (red) and PDMAEMA (black) normalised to the molar concentrations of repeating units. The pH 6 sample of the block copolymer has been normalised to the concentration of PDPA and the pH 8 and 10 samples have been normalised to the concentration of repeating units of PDMAEMA. To facilitate comparison, the block copolymer was also normalised to PDMAEMA (empty symbol).

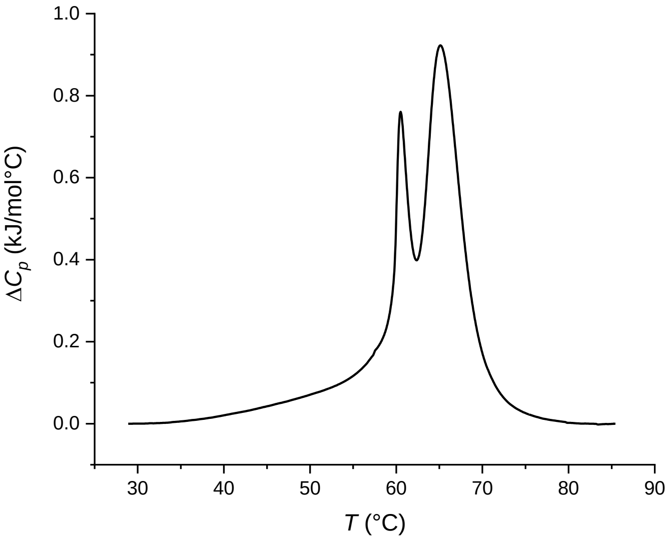

Figure S12. Thermogram of phosphate-buffered PDMAEMA-*b*-PDPA at pH 8.0. The enthalpy is normalised to the concentration of DMAEMA repeating units.

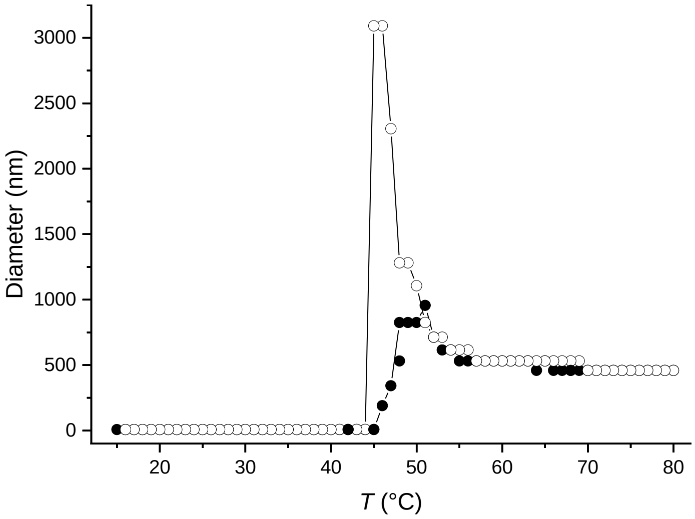

Figure S13. The intensity-average diameter of PDPA particles in the presence of 20 mM citrate as a function of temperature. Heating run (filled symbol) and the cooling run (empty symbol). pH of the solution was 5.6.

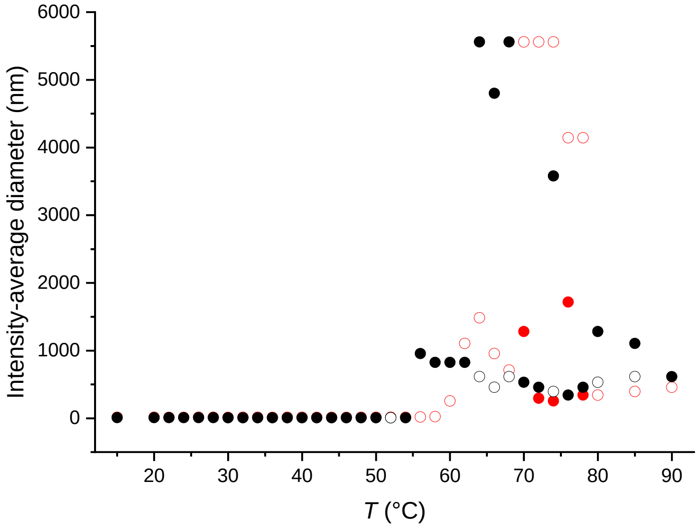

Figure S14. The apparent intensity-average diameters of PDMAEMA homopolymer (black) at pH 8.2 and PDMAEMA-*b*-PDPA (red) at pH 8.0 as a function of temperature. At some temperatures, the size distributions had two maxima, which have both been depicted – one with an empty symbol and one with a filled symbol.

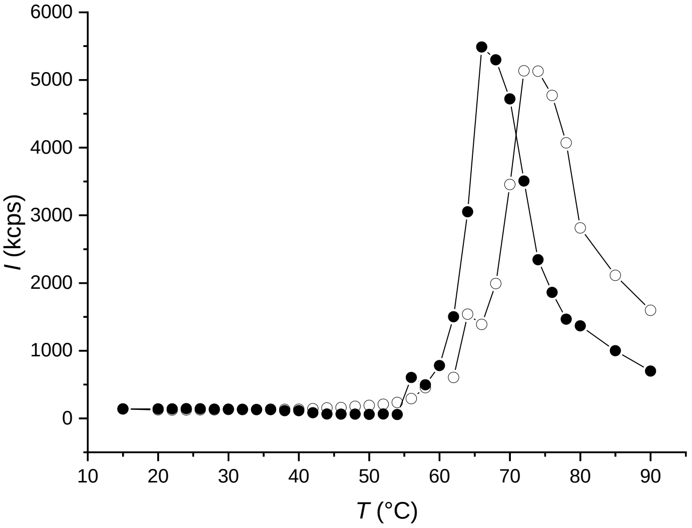

Figure S15. The scattering intensities of PDMAEMA (●) at pH 8.2 and PDMAEMA-*b*-PDPA (○) at pH 8.0.

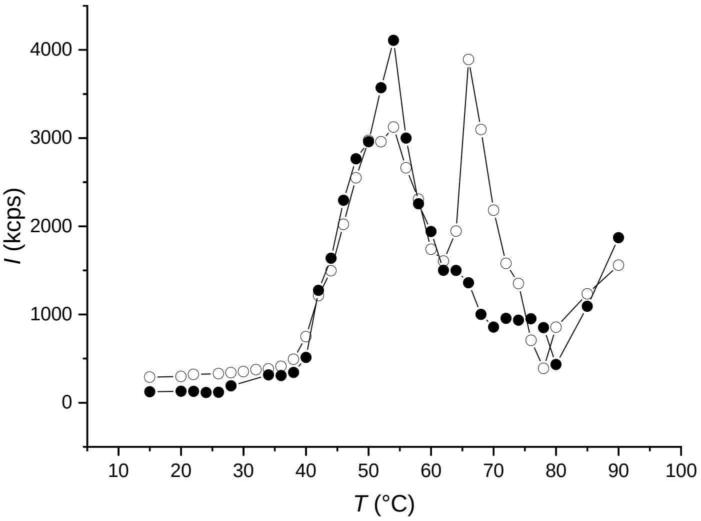

Figure S16. The scattering intensities of PDMAEMA (●) at pH 10.0 and PDMAEMA-*b*-PDPA (○) at pH 9.9. The lines are a guide for the eye.
